# Supplementary material for: Evidence-based intervention to reduce avoidable hospital admissions in care home residents (the Better Health in Residents in Care Homes (BHiRCH) study): protocol for a pilot cluster randomised trial
Source: BMJ Open. 2019 May 27;9(5):e026510. doi: 10.1136/bmjopen-2018-026510 (PMC6538003; doi:10.1136/bmjopen-2018-026510)
Supplement: Author's manuscript [file bmjopen-2018-026510.draft_revisions.pdf]

BMJ Open is committed to open peer review. As part of this commitment we make the peer review history of every article we publish publicly available.

When an article is published we post the peer reviewers' comments and the authors' responses online. We also post the versions of the paper that were used during peer review. These are the versions that the peer review comments apply to.

The versions of the paper that follow are the versions that were submitted during the peer review process. They are not the versions of record or the final published versions. They should not be cited or distributed as the published version of this manuscript.

BMJ Open is an open access journal and the full, final, typeset and author-corrected version of record of the manuscript is available on our site with no access controls, subscription charges or pay-per-view fees (http://bmjopen.bmj.com).

If you have any questions on BMJ Open's open peer review process please email info.bmjopen@bmj.com

BMJ Open

An evidence-based intervention to reduce avoidable hospital admissions in care home residents (the Better Health in Residents in Care Homes- BHiRCH study); protocol for a pilot cluster randomised trial

Journal:	*BMJ Open*
Manuscript ID	bmjopen-2018-026510
Article Type:	Protocol
Date Submitted by the Author:	05-Sep-2018

Complete List of Authors:	Sampson, Elizabeth; University College London, Division of Psychiatry<br>Feast, Alexandra; University College London, Division of Psychiatry<br>Blighe, Alan; University of Bradford, Bradford Dementia Group<br>Froggatt, Katherine ; Lancaster University, Faculty of Health and Medicine<br>Hunter, Rachael; University College London, Research Dept of Primary Care and Population Health<br>Marston, Louise; University College London,<br>McCormack, Brendan; Queen Margaret University, Nursing<br>Nurock, Shirley; University College London, Division of Psychiatry<br>Panca, Monica; University College London, Primary Care and Population Health<br>Powell, Catherine; University of Bradford, Bradford Dementia Group<br>Rait, Greta; University College London, Primary Care<br>Robinson, Louise; Institute for Health and Society, Newcastle University<br>Woodward-Carlton, Barbara; University of Bradford, Bradford Dementia Group<br>Young, John; University of Leeds, Academic Unit of Elderly Care and Rehabilitation<br>Downs, Murna; University of Bradford, Bradford Dementia Group

Keywords:	GERIATRIC MEDICINE, Urinary tract infections < UROLOGY, Respiratory infections < THORACIC MEDICINE, Heart failure < CARDIOLOGY, Dementia < NEUROLOGY, PRIMARY CARE

SCHOLARONE™
Manuscripts

An evidence-based intervention to reduce avoidable hospital admissions in care home residents (the Better Health in Residents in Care Homes-BHiRCH study); protocol for a pilot cluster randomised trial

Elizabeth L Sampson,[1,2] Alexandra Feast,[1] Alan Blighe,[3] Katherine Froggatt,[4] Rachel Hunter,[5] Louise Marston,[5] Brendan McCormack,[6] Shirley Nurock,[1] Monica Panca, Catherine Powell,[3] Greta Rait,[5] Louise Robinson,[7] Barbara Woodward-Carlton,[3] John Young,[8] Murna Downs[3]

Corresponding author
Elizabeth L. Sampson
UCL Division of Psychiatry
6th floor, Wing B, Maple House
149 Tottenham Court Road
London
W1T 7NF
02076799500/Internal 09500
e.sampson@ucl.ac.uk

[1] Marie Curie Palliative Care Research Department, Division of Psychiatry, University College London, W1T 7NF, UK.

[2] Barnet Enfield and Haringey Mental Health Trust Liaison Psychiatry Team, North Middlesex University Hospital, London, N18 1QX, UK.

[3] Centre for Applied Dementia Studies, University of Bradford, Bradford, BD7 1DP, UK.

[4] International Observatory on End of Life Care, Lancaster University, Lancaster, LA1 4QT, UK.

[5] Department of Primary Care and Population Health and Priment Clinical Trials Unit, University College London, London, NW3 2PF, UK.

[6] Divisions of Nursing, Occupational Therapy & Arts Therapies, School of Health Sciences, Queen Margaret University, Edinburgh, EH21 6UU, UK.

[7] Newcastle University Institute for Ageing and Institute for Health & Society, Newcastle University, Newcastle upon Tyne, NE4 5PL, UK.

[8] Academic Unit of Elderly Care and Rehabilitation, University of Leeds, Bradford Institute for Health Research, Bradford, BD9 6RJ, UK.

Word count: 4000

For peer review only

ABSTRACT (300 words)

Introduction: Acute hospital admission is distressing for care home residents. Ambulatory Care Sensitive Conditions (ACSCs), such as respiratory and urinary tract infections, are conditions that can cause unplanned hospital admission but may have been avoidable with timely detection and intervention in the community. The Better Health in Residents in Care Homes (BHiRCH) programme has feasibility tested and will pilot a multi-component intervention to reduce these avoidable hospital admissions. The BHiRCH intervention comprises an early warning tool for noting changes in resident health, a care pathway (clinical guidance and decision support system) and a structured method for communicating with primary care, adapted for use in the care home. We use practice development champions to support implementation and embed changes in care.

Methods and analysis: Cluster randomised pilot trial to test study procedures and indicate whether a further definitive trial is warranted. Fourteen care homes with nursing (nursing homes) will be randomly allocated to intervention (delivered at nursing home level) or control groups. Two nurses from each home become Practice Development Champions trained to implement the intervention, supported by a Practice Development Support Group. Data will be collected for 3 months pre-intervention, monthly during the 12-month intervention and one month after. Individual-level data includes resident, care partner and staff demographics, resident functional status, service use and quality of life (for health economic analysis) and the extent to which staff perceive the organisation supports person centred care. System-level data includes primary and secondary health services contacts i.e. general practitioner and hospital admissions. Process evaluation assesses intervention acceptability, feasibility, fidelity, ease of implementation in practice and study procedures i.e. consent and recruitment rates.

Ethics and dissemination: Approved by Research Ethics Committee and the UK Health Research Authority. Findings will be disseminated via academic and policy conferences, peer reviewed publications and social media e.g. Twitter.

Registration ISRCTN: 74109734 https://doi.org/10.1186/ISRCTN74109734

For peer review only - http://bmjopen.bmj.com/site/about/guidelines.xhtml

ARTICLE SUMMARY

Strengths and limitations of this study

- We are testing a complex intervention that has been developed to reduce avoidable acute hospital admissions in nursing home residents. The intervention has been developed in collaboration with key stakeholders, taking into account the complexities of implementing enhanced practice in this setting.
- A strength of the intervention is that implementation is led in the care home by Practice Development Champions, supported by Practice Development Support Group.
- We will assess feasibility of recruitment and outcome data collection at individual and system level and also consider how well the intervention was implemented in practice.
- This is a pilot study that will indicate whether a further study is warranted but will not give data on the effectiveness of the intervention.

For peer review only

INTRODUCTION

Background

Currently in the United Kingdom (UK) 421,100 people aged over 65 years live in residential care, including care homes with nursing (referred to subsequently in this paper as "nursing homes"). [1] Older people living in nursing homes have increasing levels of comorbidity, [2-4] frailty, and physical health needs. [5] Ambulatory Care Sensitive Conditions (ACSCs) are conditions that can lead to unplanned hospital admissions that may been have avoidable or manageable by timely access to medical care in the community. [6] The conditions include: angina, asthma, cellulitis, chronic obstructive pulmonary disease (COPD), congestive heart failure (CHF), dehydration, diabetes mellitus, gastroenteritis, epilepsy, hypertension, hypoglycaemia, urinary tract infections (UTI), pneumonia, severe ear, nose, and throat infections. [7]

In the UK, ACSCs account for one sixth of hospital admissions. [8] Our ageing population has led to a 40% increase in admissions between 2001 and 2011, [9] and all-cause hospital admissions from nursing homes rose by 63% between 2011 and 2015. [10] Four ACSCs contribute to a large proportion of hospitalisations from nursing homes: respiratory infections [11] [12-14]; acute exacerbation of chronic heart failure [15] [16]; urinary tract infections [13] [17]; and dehydration [13] and may underlie other problems such as falls and delirium.

As well as causing distress to residents, their families and staff, hospitalisation is expensive for the health and social care system. Hospital admission increases the risk of decline in functional ability, delirium, adverse events and prolonged stays. [18] [19] Areas with many nursing homes tend to have higher rates of unplanned hospital admission in the over-75 age group. [20] The King's Fund [8] and British Geriatrics Society [2] have raised concerns about the quality of healthcare provision to nursing homes. The UK National Health Service (NHS) [21] has made reducing avoidable hospital admission from nursing homes a policy imperative.

A number of interventions have been developed to address avoidable hospital admissions. These fall broadly into two categories- multicomponent interventions (implementation of a range of tools) and single component interventions (predominantly advance care planning or single disease care pathways, e.g. pneumonia). Multicomponent interventions show significant reductions in avoidable admissions. [22] [23] [24] [25] [26] Key characteristics of these include enhancing knowledge and skills of nursing home staff, [27] clinical guidance and decision-support tools (care pathways), engaging with families, [28] and specialist input from geriatricians or nurse practitioners. [29] In addition, research highlights the importance of collaborative development of interventions with nursing home staff, [30] residents and families,[28] considering implementation support [16] and using local champions.

For peer review only - http://bmjopen.bmj.com/site/about/guidelines.xhtml

The intervention with the strongest evidence base is "INTERACT" (Interventions to Reduce Acute Care Transfers). This complex intervention, developed and implemented in the USA, aims to detect and diagnose a range of medical conditions in residents recently discharged from hospital to skilled nursing facilities to reduce readmissions. It comprises a quality improvement programme focussing on the management of acute changes in residents' condition: [16]

- Communication tools, e.g. Stop and Watch early warning tool and SBAR (Situation Background Assessment Response) structured communication with primary care tool
- Care pathways or clinical tools addressing (for example) dehydration, UTI, fever and acute mental status change
- Advanced care planning (ACP), tracking and communication tools for use in every day practice in long-term care facilities.

Intervention development

In the preliminary stages of the Better Health in Residents of Care Homes (BHiRCH) programme we worked with stakeholders including staff and our carer reference panel (CRP), to develop and adapt the INTERACT tools for use in the UK (Blighe et al. in preparation). We identified current UK care pathways for ACSCs in nursing homes for our four key conditions (respiratory infections, acute exacerbation of chronic heart failure, urinary tract infections and dehydration) and adapted them in consensus co-design workshops with staff and family carers.

We conducted a rapid research review and semi-structured interviews to understand the optimal approach to enhance skills and knowledge in nursing home staff. The Promoting Action in Health Services (PARiHS) framework was used to co-design implementation support and guidance. [31] We conducted a feasibility and acceptability study in two nursing homes in the north of England between October 2016 and January 2017 to optimise the intervention and its implementation.

Aim and objectives

The aim of this pilot trial is to indicate whether a definitive study is warranted.

Primary objective: to indicate whether the intervention is acceptable and feasible.

Secondary objectives:

1. Establish whether consent procedures facilitate the collection of sufficient individual-level data;
2. Assess the effectiveness of the implementation strategy;
3. Assess intervention fidelity;
4. Assess the level of nursing home staff engagement with the intervention;
5. Explore whether the intervention would be sustainable outside the context of a trial;

For peer review only - http://bmjopen.bmj.com/site/about/guidelines.xhtml

6. Measure completeness of data collection, documentation, return rate of questionnaires and assess potential primary and secondary outcomes for a definitive trial:
7. Asses the feasibility of collecting data for economic evaluation.

METHODS AND ANALYSIS

Trial design

A pilot cluster randomised trial in nursing homes with a process evaluation. The intervention aims to deliver enhanced "usual care", with specifically developed tools that formalise current good practice, delivered by existing staff. The nursing home is the unit of allocation and intervention.

Study population

Nursing homes

We will recruit 14 nursing homes (8 in West Yorkshire and 6 in London) with adequate staffing to implement the intervention and support research activities. Nursing homes under investigation by the Care Quality Commission (the UK body responsible for assuring quality of care) will not be recruited. Nursing homes will be identified via local Clinical Research Networks, and the ENRICH (Enabling Research in Care Homes) Network. We will gain written permission from the manager, or regional manager, or owner. Our intervention will be implemented at nursing home level so individual consent will not be required to receive the intervention.

Individual participants

All English-speaking staff and residents over the age of 65 and their care partners (family members or friends) will be invited to participate in the collection of individual level data until we have recruited approximately 20 residents and staff from each nursing home. Residents receiving end of life care, who are under 65, non-English speaking or have stated they do not wish to be involved in research will be excluded. No further data will be collected from the care partner if the resident dies during the follow up period.

Sample size and selection

This is a pilot study to inform the sample size calculation for a definitive trial, so no formal sample size calculation was conducted. Nursing homes will be purposively selected to include a range of providers (large and small chains, independent providers), urban, suburban and rural location and size. Nursing homes will be randomised prior to the intervention starting; 4 in West Yorkshire and 3 in Greater London (7 total) to the intervention and 4 in Yorkshire and 3 in Greater London (7 total) to "usual care" stratified by location, by Priment Clinical Trials Unit (CTU) using a randomisation list drawn up by an independent statistician.

For peer review only - http://bmjopen.bmj.com/site/about/guidelines.xhtml

Consent procedures

Residents

A member of the nursing home staff will identify potentially eligible residents. If necessary, the research team will conduct a capacity assessment with respect to the participation in this trial, adhering to the Mental Capacity Act (2005).

If the resident lacks capacity to consent, we will use a personal consultee (friend or family), or if none is available, a professional consultee. This will be a member of health or social care staff with a professional relationship to the resident but no connection with the project. If a resident loses capacity during the study a consultee (either personal or professional) will be found. During the study we will consider process or ongoing consent, checking willingness to participate with the resident or consultee.

Care partners and nursing home staff

Care partners associated with residents recruited to the study and nursing home staff will be asked if they wish to answer questionnaires and/or take part in qualitative interviews and give informed consent for this.

Study procedures

The study will run for 16 months between 01/11/2017 and 03/03/2019. Posters will be placed in nursing homes and there will be sign-up sheets where potential participants can indicate their interest. We will publicise the project using established communications between the nursing home and family members such as their regular newsletters. A 'launch' event will be organised in each nursing home where the research team, members of the Carer Reference Panels and nursing home staff will explain the study and distribute recruitment literature.

Each participating nursing home will appoint a Research Facilitator to support the research team with recruitment activities, ensuring nursing home-level data collected without consent from the resident are pseudo-anonymised prior to being given to the research team. The Research Facilitator will not be involved in implementing the intervention.

INTERVENTION

This intervention is delivered by nursing home staff trained and supported by Practice Development Champions (PDCs) nominated from each nursing home by its managers, based on a person specification developed from research on facilitating implementation of research evidence (table 1).

For peer review only - http://bmjopen.bmj.com/site/about/guidelines.xhtml

Implementation support

1) Practice Development Champions workshop

A one day workshop will be delivered for the two Practice Development Champions from each nursing home by the research team comprising an introduction to the four key conditions (respiratory and urinary tract infections, dehydration and acute exacerbation of chronic heart failure) and elements of how to bring about organizational change. This will focus on: how to establish and coordinate the Practice Development Support Group of nursing home staff, care partners, and external staff (e.g. primary care professionals) who can support introduction and embedding of the change, strategies for engaging people and encouraging continued participation and strategies for gathering routinely collected data to monitor implementation. They will be given an overview of the intervention materials including the structured approach to effective communication with primary care staff. We will also explore potential changes to communication flows and recording of information about residents.

2) Ongoing implementation support

1. PDCs will be supported by a project handbook created for staff use. In addition, the *Practice Development Workbook for Nursing, Health and Social Care Teams: Resources for Health and Social Care Teams* (Dewing et al., 2014) will be provided. PDCs can access peer support on the study website and monthly telephone support from the research team.
2. PDCs will select members of a Practice Development Support Group to support their work in the nursing home. This Quality Collaborative approach[32] involves diverse stakeholders working together to close the gap between actual and potential practice. The group will support the PDCs and intervention implementation taking into account the context at their site.

The intervention

This commences after recruitment and randomisation. There are three key components:

1. Early Warning Tool (*Stop and Watch Early Warning Tool*) http://www.pathway-interact.com/wp-content/uploads/2017/04/148604-Stop-and-Watch-v4_0.pdf
2. *Care Pathway* (clinical guidance and decision support system)
3. Structured method for communicating with primary care (*SBAR*)

1. *Stop and Watch Early Warning Tool*: Care assistants or nurses will use this when they or anyone else in the nursing home (including other staff and care partners) notices a change in a resident, at latest by the end of the shift. They will circle observed changes, notifying the nurse and giving them the completed tool to be placed in the nursing home records.
2. *Care Pathway*: this is a two-step clinical guidance and decision support system. The initial "Primary" assessment comprises screening questions with the potential to trigger a more

For peer review only - http://bmjopen.bmj.com/site/about/guidelines.xhtml

detailed "Secondary" assessment. If the Primary or Secondary Assessment result is ambiguous, the *Care Pathway* will be administered at 6-hour intervals, until concerns have resolved and/or appropriate intervention has been instigated. The nurse will record the outcome of the Primary and Secondary Assessment and their care plan in the residents' care records and make a clinical decision about the next course of action which may include direct further monitoring using the *Stop and Watch Early Warning Tool*, initiating treatment in the nursing home, or if the assessment indicates a potential diagnosis or immediate concern, communication with primary care using the *SBAR* process. The nurse will feed back on the course of action to relevant staff on each shift, domestic staff and care partners, as appropriate. Copies of the completed *Care Pathway* will be kept with the resident's record.

3. *The SBAR (Situation, Background, Assessment, and Recommendation) method:* A structured method for communicating critical information to primary care. The nurse uses this when they want primary care input for one of their residents after the *Care Pathway* indicates a risk of decline. Before calling, the nurse will organise the briefing information on paper using the four elements (Situation, Background, Assessment and Recommendation).

Usual care group

Participants in nursing homes randomised to control arms will receive usual care according to existing local policy and practice. All medications and treatments will be permitted.

Study measures

We will collect data in three domains; 1) individual level data on nursing home residents, their care partners and staff, 2), system-level data, and 3) process data.

Individual-level data

These will be collected from staff, care partners or residents who have given informed consent or from residents for whom we have obtained agreement from a consultee. These data includes resident, care partner and staff demographics, assessment of resident functional status (Barthel index[33]) and resident service use and quality of life to inform health economic analysis (Client Services Receipt Inventory[34] (CSRI), EQ-5D-5L[35])). Staff education, role and the extent to which they perceive the organisation to support person centred care (Organisational Support for Person-centred Care[36] (P-CAT)) and Nurse Ratings of Communication with Primary Care Questionnaire[37] will be gathered to understand the context (table 2).

For peer review only - http://bmjopen.bmj.com/site/about/guidelines.xhtml

System-level data

These will be collected from the nursing home using existing record systems. Data will be pseudo-anonymised and provided by the nursing home manager or Research Facilitator. We will document the total number of contacts with GPs, ambulances, A&E visits, deaths, staff turnover and the number of nursing home beds available to new residents in the previous month. We will document all hospital admissions and select a sample of 30 of these (where we have individual consent from a resident to access their health records), purposively selected to represent a range of underlying ACSCs diagnoses. We will explore the "avoidability" of these with the Structured Implicit Record Review (SIRR) tool. [38] This facilitates comparison by two clinical experts of selected content in residents' nursing home records to assess whether the admission was "avoidable".

Process evaluation

We will examine how the intervention is implemented in practice, collecting information on the number of "Stop and Watch" Early Warning Tools completed, who noticed the change in the resident's health status, who completed the form, and actions that occurred after the form was completed; number of primary and secondary assessments, and outcome of assessments including the number of residents who underwent further monitoring, had their treatment initiated in the nursing home, or were referred to primary care. We will monitor intervention fidelity noting where nursing homes make amendments to the structure or content of the care pathway. PDCs will have a monthly support phone call with the research team to reflect on their activities and achievements which we will document. They will keep an activity log of support provided by Practice Development Support Groups to document the level of facilitation required to support the implementation process. To understand the effectiveness of study procedures we will collect data on consent and recruitment rates, the numbers of care partners who wish to be involved in the resident's care, assess completeness of outcome measures, data collection and return rate of questionnaires.

We will conduct 20 semi-structured interviews (30 to 45 minutes each) with 5 nursing home managers, 5 nurses, 5 care assistants and 5 care partners from the 7 intervention homes. We will explore participants' views on the effectiveness of the intervention in preventing avoidable hospital admissions and their experiences of implementing the intervention. Care partners will be purposively sampled to ensure a range of gender, age and types of family carer. All participants will give informed consent, including for recording of their interviews.

Data management

All data will be entered onto paper case report forms (CRFs) and then into an encrypted password protected database in accordance with the UK Data Protection Act and General Data Protection Regulation (GDPR). CRFs will not bear the participant's name but a pseudo-anonymised

For peer review only - http://bmjopen.bmj.com/site/about/guidelines.xhtml

identification number. To maintain confidentiality the Research Facilitator will remove the name of the participant from completed Stop and Watch Early Warning Tool and Care Pathway Primary and Secondary Assessment Forms and replace this with an ID number. Prior to analysis a standardised process for database lock will be followed.

Randomisation

The SAS statistical programme will be used for randomisation. Blinding is not feasible for research staff collecting data, but statisticians and health economists will be blinded to allocation. The randomisation variable will be supplied to them unlabelled, and main analysis completed using this.

Data analysis plan

We shall follow CONSORT guidelines for reporting randomised trials. Given this is a pilot study, analyses will be mainly descriptive focussing on recruitment, participant characteristics, other baseline and outcome variables, loss to follow-up and tabulation of serious adverse events. We shall compare rates of hospital admission for ACSCs and other outcomes between the control and intervention groups and calculate 95% confidence intervals. These, along with estimates of standard deviations of other outcome measures and intraclass correlation coefficients (ICCs) will inform the sample size calculation for a full trial. We will summarise completeness of data collection on all outcome measures and, for questionnaires, describe distributions and response rates.

Economic evaluation

Analyses will conform to accepted economic evaluation methods (NICE, 2008). We will calculate costs associated with the intervention, including the cost of enhancing staff's knowledge and skills and resources associated with implementation. Resource use associated with hospital admissions, primary care and other NHS and social care costs will be collected using the CSRI. Costs will be reported from an NHS/PSS, government and societal perspective. We will assess the feasibility of calculating quality adjusted life years (QALYs) for residents, using the EQ-5D- 5L. In the societal level analysis, a calculation of carers' QALYs will be included. We will provide an initial estimate of the incremental mean cost per QALY gained in intervention compared to control homes. Cost-effectiveness acceptability curves, showing the percentage of cases that the intervention is cost-effective over a range of values of willingness to pay for a QALY gained, will be constructed for each different costing perspective and for the different methods of calculating QALYs. We will model the lifetime costs and outcomes of the intervention compared to controls. This will involve assessing the quality of the published information available, the development of an initial model and identification of which cost and outcome components would benefit most from further research i.e. extra value of perfect information (EVPI) and extra value of partial perfect information (EVPPI) analysis.[39 40]

For peer review only - http://bmjopen.bmj.com/site/about/guidelines.xhtml

Qualitative methods

A verbatim transcript of qualitative interviews will be made and entered into qualitative analysis software (NVIVO) and key themes coded using framework analysis.[41] A sample of interviews will be analysed by two senior investigators to check levels of coding agreement with the template.

Public and patient involvement

The original research proposal was developed in collaboration with UK Dementia and Neurodegenerative Diseases Network (DeNDRoN) Patient and Public Involvement representatives (Nurock and Woodward-Carlton), who are grant co-applicants. Two Carer reference Panels (CRPs), have been created to ensure public involvement at all stages, chaired by Nurock (London) and Woodward-Carlton (Bradford). Each panel comprises up to eight family carers of people with dementia and a person living with dementia. The carers are all members of, and supported by, the Alzheimer's Society research volunteer network. They will meet at 6-monthly intervals throughout the programme, advise and work collaboratively on recruitment and consent processes, accessibility of information leaflets, data collection, interpretation and dissemination. Our CRP chairs will attend study recruitment and information events at participating care homes to publicise the trial. They provide strategic oversight by attending programme steering group and international advisory meetings. We held informal consultations with a group of seven residents living in one nursing home to inform development of study information materials for residents.

Monitoring and trial management

Our intervention is low-risk and an enhancement of usual care. We will collect data on potential serious adverse events (SAEs); those that may result in death, be life-threatening, require hospitalisation or prolongation of existing hospitalisation or result in persistent or significant disability or incapacity. These will be reported to the Chief Investigator and escalated to the research ethics committee if they are considered related to research procedures and unexpected.

A Trial Management Group meets monthly and includes individuals responsible for day-to-day study management. It will monitor all aspects of the conduct and progress of the study, ensuring adherence to the protocol and will take appropriate action to safeguard participants and trial quality. For this pilot trial it also takes on the role of a Data Monitoring Committee. The BHiRCH Programme Steering Committee includes senior members of the research team and external experts and supervises the overall programme, on behalf of NIHR and the Sponsor. An International Advisory Group (IAG) provides advice and guidance to ensure the project remains grounded in real experience and is informed by international best practice and research.

For peer review only - http://bmjopen.bmj.com/site/about/guidelines.xhtml

DISCUSSION

The key aim of our study is to pilot test an intervention to reduce avoidable acute hospital admissions for the four most common ACSCs in UK nursing home residents. Our intervention is closely aligned with current UK health policy which aims to improve healthcare provided within nursing homes and prevent distressing transitions to acute hospital care. Despite policy imperatives, little empirical research has been conducted in this area in the UK. The quality of intervention studies has varied with insufficient attention paid to methodology, particularly implementation, intervention adherence and clustering effects.

We have adapted elements of an existing intervention (INTERACT) to the UK context with stakeholder input and extensive co-design. Trials of complex interventions are often methodologically challenging but the nursing home environment brings additional issues. Each nursing home, even those that are part of large company chains, is unique in context, with a distinct culture of care and variability in practice which may be greater than that seen in clinical NHS settings. A particular strength of our intervention is that the implementation is informed by implementation science theory, tailored to the context and led by local Practice Development Champions supported by a local Practice Development Champion Support Group comprised of key stakeholders who can assist with achieving change in care practice.

We anticipate that participant recruitment, particularly residents with conditions that may lead to impaired capacity to consent to research procedures may be challenging. Additionally, nursing homes may not have the infra-structure to support collection of trial data. We plan to overcome these problems through developing the role of an existing member of the nursing home staff as a Research Facilitator and will provide reimbursement for their time and additional work required.

Our pilot study will provide data to indicate whether a fully-powered randomised controlled trial is warranted. In addition it will provide invaluable information on how to optimise the implementation of complex interventions in nursing homes.

ETHICS AND DISSEMINATION

The trial has been approved by London Queen Square Research Ethics Committee (Reference: 17/LO/1542) and the UK Health Research Authority (HRA). Some residents may have dementia or other conditions that impair their ability to give informed consent to participate in the individual data collection and we will adhere to the UK Mental Capacity Act (2005). We have an established procedure should we observe any incidents of concern and will adhere to local authority safeguarding procedures.

For peer review only - http://bmjopen.bmj.com/site/about/guidelines.xhtml

Academic dissemination will be through publication of trial results, reported as per the CONSORT guidelines in international peer-review journals and conference presentations. Regular updates will be given via social media, our website (https://www.brad.ac.uk/health/dementia/research/bhirch/) and twitter account (@BHIRCHCareHomes). We will present at congresses attended by nursing home owners and staff and will hold a national conference at the end of the study to disseminate findings and share best practice on active care for ACSCs.

For peer review only

For peer review only - http://bmjopen.bmj.com/site/about/guidelines.xhtml

Acknowledgements

The authors would like to thank the members of our carer reference panels and the Alzheimer's Society research network volunteers. We are grateful to the nursing homes that supported our feasibility study.

Authors' contributions

All authors were involved in development of the intervention and made substantial contributions to the concept and design of the study and protocol. All authors critically revised the manuscript and approved the final version.

Funding: This work was supported by the UK NIHR grant number RP-PG-0612-20010. The views expressed are those of the author(s) and not necessarily those of the NHS, the NIHR or the Department of Health and Social Care.

Competing interests statement: none declared

Ethics approval: approved by London Queen Square Research Ethics Committee (Reference: 17/LO/1542)

Provenance and peer review: not commissioned; externally peer reviewed

For peer review only

References

1. Laing and Buisson. Care of Older People UK Market Report. 28th ed, 2017.

2. British Geriatric Society. Quest for quality in care homes: British Geriatrics Society Joint Working Party Inquiry into the Quality of Healthcare Support for Older People in Care Homes: A Call for Leadership, Partnership and Quality Improvement. London: British Geriatric Society, 2011.

3. Dudman J, Meyer J. Understanding residential home issues to meet health-care needs. *British Journal of Community Nursing* 2012;17(9):434-8.

4. McDougall F, Matthews F, Kvaal K, et al. Prevalence and symptomatology of depression in older people living in institutions in England and Wales. *Age and Ageing* 2007;36(5):562-8.

5. Baylis A, Perks-Baker S. Enhanced health in care homes: Learning from experiences so far. London, 2017.

6. Carter MW, Porell FW. Vulnerable populations at risk of potentially avoidable hospitalizations: the case of nursing home residents with Alzheimer's disease. *American journal of Alzheimer's disease and other dementias* 2005;20(6):349-58. [published Online First: 2006/01/07]

7. Grabowski DC, O'Malley AJ, Barhydt NR. The costs and potential savings associated with nursing home hospitalizations. *Health affairs (Project Hope)* 2007;26(6):1753-61. doi: 10.1377/hlthaff.26.6.1753 [published Online First: 2007/11/06]

8. Tian Y, Dixon A, Gao H. Emergency hospital admissions for ambulatory care-sensitive conditions: identifying the potential for reductions: King's Fund, 2012.

9. Bardsley M, Blunt I, Davies S. Is secondary preventive care improving? Observational study of 10-year trends in emergency admissions for conditions amenable to ambulatory care. *BMJ Open* 2013;3(1) doi: 10.1136/bmjopen-2012- 002007

10. HES Analysis Team. Counts of finished admission episodes (FAEs) with an emergency admission by source of admission from 2009-10 to 2015-16. In: Health and Social Care Information Centre, ed. Leeds, 2016.

11. Purdy S, Griffin T, Salisbury C, et al. Ambulatory care sensitive conditions: terminology and disease coding need to be more specific to aid policy makers and clinicians. *Public Health* 2009;123:169-73.

12. Walsh E, Wiener J, Haber S. Potentially avoidable hospitalizations of dually eligible Medicare and Medicaid beneficiaries from nursing facility and Home- and Community-Based Services waiver programs. *Journal of the American Geriatric Society* 2012;60(5):821-9. doi: 10.1111/j.1532-5415.2012.03920.x

13. Carter M, Porell F. Vulnerable populations at risk of potentially avoidable hospitalizations: the case of nursing home residents with Alzheimer's disease. *American Journal of Alzheimer's Disease and Other Dementias* 2005;20(6):349-58.

For peer review only - http://bmjopen.bmj.com/site/about/guidelines.xhtml

14. Krüger K, Jansen K, Grimsmo A. Hospital admissions from nursing homes: Rates and reasons. *Nursing Research and Practice* 2011 doi: 10.1155/2011/247623

15. Ouslander J, Diaz S, Hain D. Frequency and diagnoses associated with 7- and 30- day readmission of skilled nursing facility patients to a nonteaching community hospital. *Journal of the American Medical Directors Association* 2011;12(3):195-203. doi: 10.1016/j.jamda.2010.02.015

16. Ouslander J, Lamb G, Tappen R. Interventions to reduce hospitalizations from nursing homes: evaluation of the INTERACT II collaborative quality improvement project. *Journal of the American Geriatric Society* 2011;59(4):745-53. doi: 10.1111/j.1532- 5415.2011.03333.x

17. Alessi C, Harker J. A prospective study of acute illness in the nursing home. *Aging* 1998;10(6):479-89.

18. Mukadam N, Sampson EL. A systematic review of the prevalence, associations and outcomes of dementia in older general hospital inpatients. *IntPsychogeriatr* 2010:1-12.

19. Watkin L, Blanchard MR, Tookman A, et al. Prospective cohort study of adverse events in older people admitted to the acute general hospital: risk factors and the impact of dementia. *IntJGeriatrPsychiatry* 2012;27(1):76-82. doi: 10.1002/gps.2693 [doi]

20. Smith P, Sherlaw-Johnson C, Ariti C, et al. Quality watch. Focus on: Hospital admissions from care homes. London: Health Foundation and Nuffield Trust, 2015.

21. National Health Service. NHS Outcomes Framework 2016 to 2017, 2016.

22. Ouslander JG, Perloe M, Givens JH, et al. Reducing potentially avoidable hospitalizations of nursing home residents: results of a pilot quality improvement project. *JAmMedDirAssoc* 2009;10(9):644-52. doi: S1525-8610(09)00248-5 [pii];10.1016/j.jamda.2009.07.001 [doi]

23. Ouslander JG, Berenson RA. Reducing unnecessary hospitalizations of nursing home residents. *NEnglJMed* 2011;365(13):1165-67. doi: 10.1056/NEJMp1105449 [doi]

24. Lisk R, Yeong K, Nasim A, et al. Geriatrician input into nursing homes reduces emergency hospital admissions. *Archives of gerontology and geriatrics* 2012;2011/11/25:331-37.

25. Codde J, Arendts G, Frankel J, et al. Transfers from residential aged care facilities to the emergency department are reduced through improved primary care services: an intervention study. *Australasian journal on ageing* 2010;2010/12/15:150-54.

26. Kane RL, Keckhafer G, Flood S, et al. The effect of Evercare on hospital use. *Journal of the American Geriatrics Society* 2003;2003/09/27:1427-34.

27. Mor V, Intrator O, Fries B. Changes in hospitalization associated with introducing the Resident Assessment Instrument. *Journal of the American Geriatric Society* 1997;45(8):1002-10.

28. Young Y, Inamdar S, Dichter B. Clinical and nonclinical factors associated with potentially preventable hospitalizations among nursing home residents in New York State. *Journal of the American Medical Directors Association* 2011;12(5):364-71. doi: 10.1016/j.jamda.2010.03.006

29. Ouslander J, Perloe M, Givens J. Reducing potentially avoidable hospitalizations of nursing home residents: results of a pilot quality improvement project. *Journal of the American Medical Directors Association* 2009;10(9):644-52. doi: 10.1016/j.jamda.2009.07.001

For peer review only - http://bmjopen.bmj.com/site/about/guidelines.xhtml

30. Olsan T. Reducing avoidable Rehospitalisations by Improving Residents' Transitions to the Nursing Home. 2011:B22.

31. Kitson AL, Rycroft-Malone J, Harvey G, et al. Evaluating the successful implementation of evidence into practice using the PARiHS framework: theoretical and practical challenges. *ImplementSci* 2008;3:1.

32. Rycroft-Malone J, Harvey G, Seers K, et al. An exploration of the factors that influence the implementation of evidence into practice. *JClinNurs* 2004;13(8):913-24.

33. Mahoney FI, Barthel DW. Functional Evaluation: The Barthel Index. *Md State Med J* 1965;14:61-5. [published Online First: 1965/02/01]

34. Beecham J, Knapp M. Costing psychiatric interventions. In: Thornicroft G, ed. Measuring Mental Helath Needs. London: Gaskell 2001:200-24.

35. Herdman M, Gudex C, Lloyd A, et al. Development and preliminary testing of the new five-level version of EQ-5D (EQ-5D-5L). *Qual Life Res* 2011;20(10):1727-36. doi: 10.1007/s11136-011-9903-x [published Online First: 2011/04/12]

36. Edvardsson D, Fetherstonhaugh D, Nay R, et al. Development and initial testing of the Person-centered Care Assessment Tool (P-CAT). *International psychogeriatrics / IPA* 2010;22(1):101-8. doi: 10.1017/S1041610209990688 [published Online First: 2009/07/28]

37. Tjia J, Mazor KM, Field T, et al. Nurse-physician communication in the long-term care setting: perceived barriers and impact on patient safety. *J Patient Saf* 2009;5(3):145-52. doi: 10.1097/PTS.0b013e3181b53f9b [published Online First: 2009/11/21]

38. Saliba D, Kington R, Buchanan J, et al. Appropriateness of the decision to transfer nursing facility residents to the hospital. *Journal of the American Geriatrics Society* 2000;2000/02/22:154-63.

39. Ades AE, Lu G, Claxton K. Expected value of sample information calculations in medical decision modeling. *Med Decis Making* 2004;24(2):207-27. doi: 10.1177/0272989X04263162 [published Online First: 2004/04/20]

40. Coyle D, Oakley J. Estimating the expected value of partial perfect information: a review of methods. *Eur J Health Econ* 2008;9(3):251-9. doi: 10.1007/s10198-007-0069-y [published Online First: 2007/07/20]

41. Ritchie J, Spencer L. Qualitative data analysis for applied policy research. In: Ryman A, Burgess AG, eds. Analysing qualitative data. London: Routledge 1994.

For peer review only - http://bmjopen.bmj.com/site/about/guidelines.xhtml

Table 1. Practice Development Champion person specification

The Practice Development Champion will:

- Be a Registered Nurse.
- Have been working in the nursing home for at least 6 months.

When selecting a Practice Development Champion we are looking for someone who:

- Has some knowledge of good practice in supporting health care and has an interest in the topic *(can demonstrate some essential knowledge of the management of the 4 conditions: chronic heart failure, respiratory infections, UTI, dehydration).*
- Knows co-workers *(has been in the organisation long enough to know the staff and how they work).*
- Knows the environment *(has some insight into the culture of the setting).*
- Knows the organisation *(knows their way around the organisation, e.g. who's who, policies in place, decision-making structures).*
- Possesses effective communication skills *(could include attributes of being open minded, being creative, has experience of managing meetings/groups, able to talk in front of groups).*
- Is self-aware and resilient *(has insight into their support needs, but is also not afraid of challenge/conflict; willing to engage in own professional development).*
- Is reliable and dependable *(has time they can dedicate to this work [in writing from their manager]; carries through with responsibilities, meets deadlines or negotiates otherwise; is not intending to be on extended leave during intervention period).*
- Is respected by co-workers (*has a good relationship with co-workers which means they will be listened to with respect to new ideas*).

These criteria are ESSENTIAL and are NOT listed in a hierarchy/order of importance, i.e. they are all equally important.

From Seers et al 2012 FIRE (facilitating implementation of research evidence): a study protocol http://implementationscience.biomedcentral.com/articles/10.1186/1748-5908-7-25

For peer review only - http://bmjopen.bmj.com/site/about/guidelines.xhtml

BHiRCH pilot RCT protocol paper

Table 2. Summary of data collected, outcome measures and time schedule

	Data collected and tool used	*Pre-intervention*	*Monthly*	*At 6 months only*	*Post intervention*
Resident					
Socio-demographics	Age, gender, ethnicity, marital status, highest level of education	R	-	-	-
Service use in the prior month	Client Service Receipt Inventory (CSRI) (ref). Calculates service and total care costs	R	R	R	R
Functional status	The Barthel Index [33]	R	-	R	R
Resident quality of life-self rated	EQ-5D-5L (ref) self-rated health index and visual analogue scale of current health state.	P	-	P	P
Resident quality of life-proxy rated	EQ-5d-Proxy (ref) care partner or staff member view of the resident's QoL	CP/S	-	CP/S	CP/S
Care partner					
Socio-demographics	Age, gender, ethnicity, marital status, years of schooling, highest level of education.	CP	-	CP	CP
Quality of life	EQ-5D-5L EuroQol (1990)	CP	-	CP	CP
Preferred role	How much and how they like to be involved in the residents care	CP	-	-	-
Staff					
Staff socio-demographics	Age, gender, ethnicity, number of years of education,	R	-	-	-
Staff work characteristics	Highest qualification, role in care home, length of service, shift pattern, first language	R	-	-	-
Organizational support for person-centered care	The Person-Centred Care Assessment Tool (P-CAT) (ref)	S	-	S	S
Communication with primary care	Nurse-GP Communication Needs Assessment Questionnaire	S	-	S	S
Perceived knowledge and skills for early detection in changes in health	Developed from feasibility study. Assesses key knowledge and skills needed to implement the intervention. Rated on 5-point likert scale; 1 (disagree completely) to 5 (agree completely).	S	-	S	S
**System-level data**					
Number of hospital admissions	Respiratory infections, urinary tract infections, dehydration, congestive heart failure?	S	S	S	S
"Avoidability" of admissions	Structured Implicit Record Review (SIRR; Saliba et al., 2000)	S	S	S	S
Use of Primary assessment tool	Respiratory infections, urinary tract infections, dehydration, congestive heart failure?	S	S	S	S
Use of Secondary assessment	Respiratory infections, urinary tract infections, dehydration, congestive heart failure?	S	S	S	S
Out of hours GP contacts	GP visits or telephone contact	S	S	S	S
Ambulances and hospital use	Number and length of hospital admissions (days) , A&E attendances and readmissions	S	S	S	S
Deaths in the last calendar month		S	S	S	S
Staff turnover		S	S	S	S
Care home occupancy level	Number of available beds to new residents	S	S	S	S

Measure assessed by: P, participant; CP, care partner; R researcher; S, care home staff.

For peer review only - http://bmjopen.bmj.com/site/about/guidelines.xhtml

Figure 1. Pilot trial Flow Chart

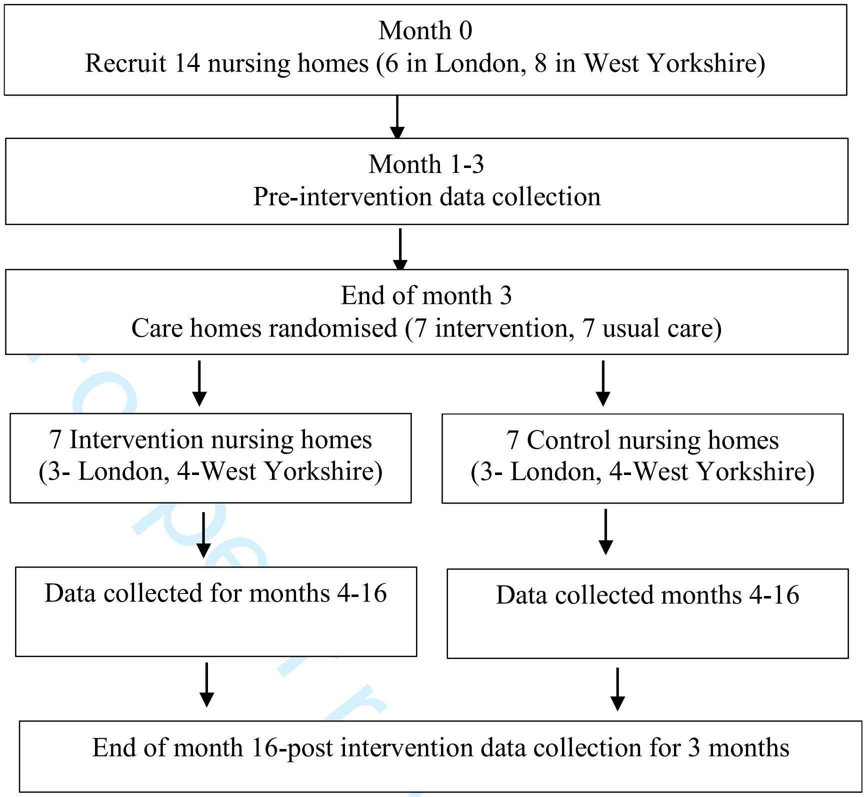

For peer review only - http://bmjopen.bmj.com/site/about/guidelines.xhtml

BHiRCH
intervention protocol-paper

SPIRIT 2013 Checklist: Recommended items to address in a clinical trial protocol and related documents*

Section/item	Item No	Description
**Administrative information**		
Title	1	Descriptive title identifying the study design, population, interventions, and, if applicable, trial acronym -yes
Trial registration	2a	Trial identifier and registry name. If not yet registered, name of intended registry-yes
	2b	All items from the World Health Organization Trial Registration Data Set-on protocol
Protocol version	3	Date and version identifier-yes uploaded
Funding	4	Sources and types of financial, material, and other support-yes
Roles and responsibilities	5a	Names, affiliations, and roles of protocol contributors-yes
	5b	Name and contact information for the trial sponsor-on protocol
	5c	Role of study sponsor and funders, if any, in study design; collection, management, analysis, and interpretation of data; writing of the report; and the decision to submit the report for publication, including whether they will have ultimate authority over any of these activities-on protocol
	5d	Composition, roles, and responsibilities of the coordinating centre, steering committee, endpoint adjudication committee, data management team, and other individuals or groups overseeing the trial, if applicable (see Item 21a for data monitoring committee)-yes
**Introduction**		
Background and rationale	6a	Description of research question and justification for undertaking the trial, including summary of relevant studies (published and unpublished) examining benefits and harms for each intervention-yes
	6b	Explanation for choice of comparators-yes
Objectives	7	Specific objectives or hypotheses-yes

For peer review only

Trial design	8	Description of trial design including type of trial (eg, parallel group, crossover, factorial, single group), allocation ratio, and framework (eg, superiority, equivalence, noninferiority, exploratory)-yes

Methods: Participants, interventions, and outcomes

Study setting	9	Description of study settings (eg, community clinic, academic hospital) and list of countries where data will be collected. Reference to where list of study sites can be obtained-yes
Eligibility criteria	10	Inclusion and exclusion criteria for participants. If applicable, eligibility criteria for study centres and individuals who will perform the interventions (eg, surgeons, psychotherapists)-yes
Interventions	11a	Interventions for each group with sufficient detail to allow replication, including how and when they will be administered-yes referenced
	11b	Criteria for discontinuing or modifying allocated interventions for a given trial participant (eg, drug dose change in response to harms, participant request, or improving/worsening disease)- not relevant
	11c	Strategies to improve adherence to intervention protocols, and any procedures for monitoring adherence (eg, drug tablet return, laboratory tests)-yes
	11d	Relevant concomitant care and interventions that are permitted or prohibited during the trial-yes
Outcomes	12	Primary, secondary, and other outcomes, including the specific measurement variable (eg, systolic blood pressure), analysis metric (eg, change from baseline, final value, time to event), method of aggregation (eg, median, proportion), and time point for each outcome. Explanation of the clinical relevance of chosen efficacy and harm outcomes is strongly recommended-yes
Participant timeline	13	Time schedule of enrolment, interventions (including any run-ins and washouts), assessments, and visits for participants. A schematic diagram is highly recommended (see Figure)-yes
Sample size	14	Estimated number of participants needed to achieve study objectives and how it was determined, including clinical and statistical assumptions supporting any sample size calculations-yes
Recruitment	15	Strategies for achieving adequate participant enrolment to reach target sample size-yes

Methods: Assignment of interventions (for controlled trials)

Allocation:

Sequence generation	16a	Method of generating the allocation sequence (eg, computer-generated random numbers), and list of any factors for stratification. To reduce predictability of a random sequence, details of any planned restriction (eg, blocking) should be provided in a separate document that is unavailable to those who enrol participants or assign interventions-yes
Allocation concealment mechanism	16b	Mechanism of implementing the allocation sequence (eg, central telephone; sequentially numbered, opaque, sealed envelopes), describing any steps to conceal the sequence until interventions are assigned-not relevant
Implementation	16c	Who will generate the allocation sequence, who will enrol participants, and who will assign participants to interventions-yes
Blinding (masking)	17a	Who will be blinded after assignment to interventions (eg, trial participants, care providers, outcome assessors, data analysts), and how-yes
	17b	If blinded, circumstances under which unblinding is permissible, and procedure for revealing a participant's allocated intervention during the trial-yes
**Methods: Data collection, management, and analysis**		
Data collection methods	18a	Plans for assessment and collection of outcome, baseline, and other trial data, including any related processes to promote data quality (eg, duplicate measurements, training of assessors) and a description of study instruments (eg, questionnaires, laboratory tests) along with their reliability and validity, if known. Reference to where data collection forms can be found, if not in the protocol-yes
	18b	Plans to promote participant retention and complete follow-up, including list of any outcome data to be collected for participants who discontinue or deviate from intervention protocols-yes
Data management	19	Plans for data entry, coding, security, and storage, including any related processes to promote data quality (eg, double data entry; range checks for data values). Reference to where details of data management procedures can be found, if not in the protocol-yes
Statistical methods	20a	Statistical methods for analysing primary and secondary outcomes. Reference to where other details of the statistical analysis plan can be found, if not in the protocol-yes
	20b	Methods for any additional analyses (eg, subgroup and adjusted analyses)-yes
	20c	Definition of analysis population relating to protocol non-adherence (eg, as randomised analysis), and any statistical methods to handle missing data (eg, multiple imputation)-yes

Methods: Monitoring

Data monitoring	21a	Composition of data monitoring committee (DMC); summary of its role and reporting structure; statement of whether it is independent from the sponsor and competing interests; and reference to where further details about its charter can be found, if not in the protocol. Alternatively, an explanation of why a DMC is not needed-yes
	21b	Description of any interim analyses and stopping guidelines, including who will have access to these interim results and make the final decision to terminate the trial-not relevant
Harms	22	Plans for collecting, assessing, reporting, and managing solicited and spontaneously reported adverse events and other unintended effects of trial interventions or trial conduct-yes
Auditing	23	Frequency and procedures for auditing trial conduct, if any, and whether the process will be independent from investigators and the sponsor-in protocol

Ethics and dissemination

Research ethics approval	24	Plans for seeking research ethics committee/institutional review board (REC/IRB) approval-yes
Protocol amendments	25	Plans for communicating important protocol modifications (eg, changes to eligibility criteria, outcomes, analyses) to relevant parties (eg, investigators, REC/IRBs, trial participants, trial registries, journals, regulators)-yes
Consent or assent	26a	Who will obtain informed consent or assent from potential trial participants or authorised surrogates, and how (see Item 32)-yes
	26b	Additional consent provisions for collection and use of participant data and biological specimens in ancillary studies, if applicable-not relevant
Confidentiality	27	How personal information about potential and enrolled participants will be collected, shared, and maintained in order to protect confidentiality before, during, and after the trial-yes
Declaration of interests	28	Financial and other competing interests for principal investigators for the overall trial and each study site-yes
Access to data	29	Statement of who will have access to the final trial dataset, and disclosure of contractual agreements that limit such access for investigators-yes
Ancillary and post-trial care	30	Provisions, if any, for ancillary and post-trial care, and for compensation to those who suffer harm from trial participation-in protocol

For peer review only

Dissemination policy	31a	Plans for investigators and sponsor to communicate trial results to participants, healthcare professionals, the public, and other relevant groups (eg, via publication, reporting in results databases, or other data sharing arrangements), including any publication restrictions-yes
	31b	Authorship eligibility guidelines and any intended use of professional writers-not relevant
	31c	Plans, if any, for granting public access to the full protocol, participant-level dataset, and statistical code-not relevant
**Appendices**		
Informed consent materials	32	Model consent form and other related documentation given to participants and authorised surrogates-in protocol
Biological specimens	33	Plans for collection, laboratory evaluation, and storage of biological specimens for genetic or molecular analysis in the current trial and for future use in ancillary studies, if applicable-not relevant

*It is strongly recommended that this checklist be read in conjunction with the SPIRIT 2013 Explanation & Elaboration for important clarification on the items. Amendments to the protocol should be tracked and dated. The SPIRIT checklist is copyrighted by the SPIRIT Group under the Creative Commons "Attribution-NonCommercial-NoDerivs 3.0 Unported" license.

For peer review only

BMJ Open

An evidence-based intervention to reduce avoidable hospital admissions in care home residents (the Better Health in Residents in Care Homes- BHiRCH study); protocol for a pilot cluster randomised trial

Journal:	*BMJ Open*
Manuscript ID	bmjopen-2018-026510.R1
Article Type:	Protocol
Date Submitted by the Author:	25-Jan-2019

Complete List of Authors:	Sampson, Elizabeth; University College London, Division of Psychiatry<br>Feast, Alexandra; University College London, Division of Psychiatry<br>Blighe, Alan; University of Bradford, Bradford Dementia Group<br>Froggatt, Katherine ; Lancaster University, Faculty of Health and Medicine<br>Hunter, Rachael; University College London, Research Dept of Primary Care and Population Health<br>Marston, Louise; University College London,<br>McCormack, Brendan; Queen Margaret University, Nursing<br>Nurock, Shirley; University College London, Division of Psychiatry<br>Panca, Monica; University College London, Primary Care and Population Health<br>Powell, Catherine; University of Bradford, Bradford Dementia Group<br>Rait, Greta; University College London, Primary Care<br>Robinson, Louise; Institute for Health and Society, Newcastle University<br>Woodward-Carlton, Barbara; University of Bradford, Bradford Dementia Group<br>Young, John; University of Leeds, Academic Unit of Elderly Care and Rehabilitation<br>Downs, Murna; University of Bradford, Bradford Dementia Group

**Primary Subject Heading**:	Geriatric medicine
Secondary Subject Heading:	Nursing
Keywords:	GERIATRIC MEDICINE, Urinary tract infections < UROLOGY, Respiratory infections < THORACIC MEDICINE, Heart failure < CARDIOLOGY, Dementia < NEUROLOGY, PRIMARY CARE

SCHOLARONE™
Manuscripts

An evidence-based intervention to reduce avoidable hospital admissions in care home residents (the Better Health in Residents in Care Homes-BHiRCH study); protocol for a pilot cluster randomised trial

Elizabeth L Sampson,[1,2] Alexandra Feast,[1] Alan Blighe,[3] Katherine Froggatt,[4] Rachael Hunter,[5] Louise Marston,[5] Brendan McCormack,[6] Shirley Nurock,[1] Monica Panca, Catherine Powell,[3] Greta Rait,[5] Louise Robinson,[7] Barbara Woodward-Carlton,[3] John Young,[8] Murna Downs[3]

Corresponding author
Elizabeth L. Sampson
UCL Division of Psychiatry
6th floor, Wing B, Maple House
149 Tottenham Court Road
London
W1T 7NF
02076799500/internal 09500
e.sampson@ucl.ac.uk

[1] Marie Curie Palliative Care Research Department, Division of Psychiatry, University College London, W1T 7NF, UK.

[2] Barnet Enfield and Haringey Mental Health Trust Liaison Psychiatry Team, North Middlesex University Hospital, London, N18 1QX, UK.

[3] Centre for Applied Dementia Studies, University of Bradford, Bradford, BD7 1DP, UK.

[4] International Observatory on End of Life Care, Lancaster University, Lancaster, LA1 4QT, UK.

[5] Department of Primary Care and Population Health and Priment Clinical Trials Unit, University College London, London, NW3 2PF, UK.

[6] Divisions of Nursing, Occupational Therapy & Arts Therapies, School of Health Sciences, Queen Margaret University, Edinburgh, EH21 6UU, UK.

[7] Newcastle University Institute for Ageing and Institute for Health & Society, Newcastle University, Newcastle upon Tyne, NE4 5PL, UK.

[8] Academic Unit of Elderly Care and Rehabilitation, University of Leeds, Bradford Institute for Health Research, Bradford, BD9 6RJ, UK.

Word count: 4000

For peer review only

BHiRCH pilot RCT protocol paper

ABSTRACT (300 words)

Introduction: Acute hospital admission is distressing for care home residents. Ambulatory Care Sensitive Conditions (ACSCs), such as respiratory and urinary tract infections, are conditions that can cause unplanned hospital admission but may have been avoidable with timely detection and intervention in the community. The Better Health in Residents in Care Homes (BHiRCH) programme has feasibility tested and will pilot a multi-component intervention to reduce these avoidable hospital admissions. The BHiRCH intervention comprises an early warning tool for noting changes in resident health, a care pathway (clinical guidance and decision support system) and a structured method for communicating with primary care, adapted for use in the care home. We use practice development champions to support implementation and embed changes in care.

Methods and analysis: Cluster randomised pilot trial to test study procedures and indicate whether a further definitive trial is warranted. Fourteen care homes with nursing (nursing homes) will be randomly allocated to intervention (delivered at nursing home level) or control groups. Two nurses from each home become Practice Development Champions trained to implement the intervention, supported by a Practice Development Support Group. Data will be collected for 3 months pre-intervention, monthly during the 12-month intervention and one month after. Individual-level data includes resident, care partner and staff demographics, resident functional status, service use and quality of life (for health economic analysis) and the extent to which staff perceive the organisation supports person centred care. System-level data includes primary and secondary health services contacts i.e. general practitioner and hospital admissions. Process evaluation assesses intervention acceptability, feasibility, fidelity, ease of implementation in practice and study procedures i.e. consent and recruitment rates.

Ethics and dissemination: Approved by Research Ethics Committee and the UK Health Research Authority. Findings will be disseminated via academic and policy conferences, peer reviewed publications and social media e.g. Twitter.

Registration ISRCTN: 74109734 https://doi.org/10.1186/ISRCTN74109734

For peer review only

For peer review only - http://bmjopen.bmj.com/site/about/guidelines.xhtml

ARTICLE SUMMARY

Strengths and limitations of this study

- We are testing a complex intervention that has been developed to reduce avoidable acute hospital admissions in nursing home residents. The intervention has been developed in collaboration with key stakeholders, taking into account the complexities of implementing enhanced practice in this setting.
- A strength of the intervention is that implementation is led in the care home by Practice Development Champions, supported by Practice Development Support Group.
- We will assess feasibility of recruitment and outcome data collection at individual and system level and also consider how well the intervention was implemented in practice.
- This is a pilot study that will indicate whether a further study is warranted but will not give data on the effectiveness of the intervention.

For peer review only

INTRODUCTION

Background

Currently in the United Kingdom (UK) 421,100 people aged over 65 years live in residential care, including care homes with nursing (referred to subsequently in this paper as "nursing homes"). [1] Older people living in nursing homes have increasing levels of comorbidity, [2-4] frailty, and physical health needs. [5] Ambulatory Care Sensitive Conditions (ACSCs) are "conditions that can lead to unplanned hospital admissions that may been have avoidable or manageable by timely access to medical care in the community". [6] The conditions include: angina, asthma, cellulitis, chronic obstructive pulmonary disease (COPD), congestive heart failure (CHF), dehydration, diabetes mellitus, gastroenteritis, epilepsy, hypertension, hypoglycaemia, urinary tract infections (UTI), pneumonia, severe ear, nose, and throat infections. [7]

In the UK, ACSCs account for one sixth of hospital admissions from all age groups. [8] Our ageing population led to a 40% increase in admissions between 2001-2011, [9] and all-cause hospital admissions from nursing homes rose by 63% between 2011-2015. [10] Four ACSCs contribute to a large proportion of hospitalisations from nursing homes: respiratory infections [11] [12-14]; acute exacerbation of chronic heart failure [15] [16]; urinary tract infections [13] [17]; and dehydration [13] and may underlie other problems such as falls and delirium.

As well as causing distress to residents, their families and staff, hospitalisation is expensive for health and social care systems. Hospital admission increases the risk of decline in functional ability, delirium, adverse events and prolonged stays. [18] [19] Areas with many nursing homes tend to have higher rates of unplanned hospital admission in the over-75 age group. [20] The King's Fund [8] and British Geriatrics Society [2] have raised concerns about the quality of healthcare provision to nursing homes. The UK National Health Service (NHS) [21] has made reducing avoidable hospital admission from nursing homes a policy imperative.

A number of interventions have been developed, falling broadly into two categories; multicomponent interventions (implementation of a range of tools) and single component interventions (predominantly advance care planning or single disease care pathways, e.g. pneumonia). Multicomponent interventions show significant reductions in avoidable admissions. [22] [23] [24] [25] [26] Key characteristics include enhancing knowledge and skills of nursing home staff, [27] clinical guidance and decision-support tools (care pathways), engaging with families, [28] and specialist input from geriatricians or nurse practitioners. [29] In addition, research highlights the importance of collaborative development of interventions with nursing home staff, [30] residents and families,[28] considering implementation support [16] and using local champions.

For peer review only - http://bmjopen.bmj.com/site/about/guidelines.xhtml

The intervention with the strongest evidence base is "INTERACT" (Interventions to Reduce Acute Care Transfers). This complex intervention, developed and implemented in the USA, aims to detect and diagnose a range of medical conditions in residents recently discharged from hospital to skilled nursing facilities and reduce readmissions. It comprises a quality improvement programme focussing on the management of acute changes in residents' condition: [16]

- Communication tools, e.g. Stop and Watch early warning tool and SBAR (Situation Background Assessment Response) structured communication with primary care tool
- Care pathways or clinical tools addressing (for example) dehydration, UTI, fever and acute mental status change
- Advanced care planning (ACP), tracking and communication tools.

Intervention development

In the preliminary stages of the Better Health in Residents of Care Homes (BHiRCH) programme we worked with stakeholders including staff and our carer reference panel (CRP), to develop and adapt the INTERACT tools for use in the UK (Blighe et al. in preparation). We identified current UK care pathways for ACSCs in nursing homes for our four key conditions (respiratory infections, acute exacerbation of chronic heart failure, urinary tract infections and dehydration) and adapted them in consensus co-design workshops with staff and care partners.

We conducted a rapid research review and semi-structured interviews to understand the optimal approach to enhance skills and knowledge in nursing home staff. The Promoting Action in Health Services (PARiHS) framework was used to co-design implementation support and guidance. [31] We conducted a feasibility and acceptability study in two nursing homes in the north of England between October 2016 and January 2017 to optimise the intervention and its implementation.

Aim and objectives

The aim of this pilot trial is to indicate whether a definitive study is warranted.

Primary objective: to indicate whether the intervention is acceptable and feasible.

Secondary objectives:

1. Establish whether consent procedures facilitate collection of sufficient individual-level data;
2. Assess the effectiveness of the implementation strategy;
3. Assess intervention fidelity;
4. Assess level of nursing home staff engagement with the intervention;
5. Explore whether the intervention would be sustainable outside the trial context;
6. Measure completeness of data collection, documentation, return rate of questionnaires and assess potential primary and secondary outcomes for a definitive trial:

For peer review only - http://bmjopen.bmj.com/site/about/guidelines.xhtml

7. Assess feasibility of collecting data for economic evaluation.

METHODS AND ANALYSIS

Trial design

A pilot cluster randomised trial in nursing homes with process evaluation. The intervention aims to deliver enhanced "usual care", with specifically developed tools that formalise current good practice, delivered by existing staff. The nursing home is the unit of allocation and intervention.

Study population

Nursing homes

We will recruit 14 nursing homes (8 in West Yorkshire and 6 in London) with adequate staffing to implement the intervention and support research activities. Nursing homes rated "inadequate" by the Care Quality Commission (UK body responsible for assuring care quality) are ineligible. Nursing homes will be identified via local Clinical Research Networks, and the ENRICH (Enabling Research in Care Homes) Network. We will gain written permission from the manager, regional manager, or owner. Our intervention will be implemented at nursing home level so individual consent is not required to receive the intervention.

Individual participants

All English-speaking staff and residents over 65 years and their care partners (family members or friends) will be invited to participate in the collection of individual level data until we have recruited approximately 20 residents and staff from each nursing home. Residents receiving end-of-life care, who are under 65, non-English speaking or have stated they do not wish to be involved in research will be excluded. No further data will be collected from the care partner if the resident dies during follow-up.

Sample size and selection

This is a pilot study to inform the sample size calculation for a definitive trial, so no sample size calculation was conducted. Nursing homes will be purposively selected including a range of providers (large and small chains, independent providers), urban, suburban and rural. Nursing homes will be randomised prior to intervention; 4 in West Yorkshire and 3 in Greater London (7 total) to the intervention and 4 in Yorkshire and 3 in Greater London (7 total) to "usual care" stratified by location, by Priment Clinical Trials Unit (CTU) using a randomisation list drawn up by an independent statistician.

For peer review only - http://bmjopen.bmj.com/site/about/guidelines.xhtml

Consent procedures

Residents

The care home manager or deputy manager will identify all potentially eligible residents. If necessary, the research team will conduct a capacity assessment with respect to the participation in this trial, adhering to the Mental Capacity Act (2005).

If the resident lacks capacity to consent, we will use a personal consultee (friend or family), or if none is available, a professional consultee. This will be a member of health or social care staff with a professional relationship to the resident but no connection with the project. If a resident loses capacity during the study a consultee (either personal or professional) will be found. During the study we will consider ongoing (process) consent, checking willingness to participate with the resident or consultee.

Care partners and nursing home staff

Care partners associated with residents who have been recruited to the study and nursing home staff will be asked if they wish to answer questionnaires and/or take part in qualitative interviews and give informed consent for this.

Study procedures

The study will run for 16 months between 01/11/2017 and 03/03/2019 (see figure 1). Posters will be placed in nursing homes and there will be sign-up sheets where potential participants can indicate interest. We will publicise the project using established communications between the nursing home and care partners such as their regular newsletters. A 'launch' event will be organised in each nursing home where the research team, members of the Carer Reference Panels and nursing home staff will explain the study and distribute recruitment literature.

Each participating nursing home will appoint a Research Facilitator to support the research team with recruitment activities, ensuring nursing home-level data collected without consent from the resident are pseudo-anonymised prior to being given to the research team. The Research Facilitator may be a care home nurse or administrator and will be supported by the research assistant allocated to the care home. They will not be involved in implementing the intervention.

INTERVENTION

This intervention is delivered by nursing home staff trained and supported by Practice Development Champions (PDCs) nominated from each intervention nursing home by its managers, based on a person specification developed from research on facilitating implementation of research evidence (table 1).

For peer review only - http://bmjopen.bmj.com/site/about/guidelines.xhtml

For peer review only

Implementation support

1) Practice Development Champions workshop

A one day workshop will be delivered for the two Practice Development Champions from each nursing home by the research team comprising an introduction to the four key conditions (respiratory and urinary tract infections, dehydration and acute exacerbation of chronic heart failure) and elements of how to bring about organizational change. This will focus on: how to establish and coordinate the Practice Development Support Group of nursing home staff, care partners, and external staff (e.g. primary care professionals) who can support introduction and embedding of the change, strategies for engaging people and encouraging continued participation and strategies for gathering routinely collected data to monitor implementation. They will be given an overview of the intervention materials including the structured approach to effective communication with primary care staff. We will also explore potential changes to communication flows and recording of information about residents.

2) Ongoing implementation support

1. PDCs will be supported by a project handbook created for staff use. In addition, the *Practice Development Workbook for Nursing, Health and Social Care Teams: Resources for Health and Social Care Teams* (Dewing et al., 2014) will be provided. PDCs can access peer support on the study website and monthly telephone support from the research team.
2. PDCs will select members of a Practice Development Support Group to support their work in the nursing home. This Quality Collaborative approach[32] involves diverse stakeholders working together to close the gap between actual and potential practice. The group will support the PDCs and intervention implementation taking into account the context at their site.
3. Monthly support phone call between practice development champions and a senior nurse researcher

The intervention

This commences after recruitment and randomisation. There are three key components, all paper based as UK care homes have variable use of electronic records:

1. Early Warning Tool (*Stop and Watch Early Warning Tool*) http://www.pathway-interact.com/wp-content/uploads/2017/04/148604-Stop-and-Watch-v4_0.pdf
2. *Care Pathway* (clinical guidance and decision support system)
3. Structured method for communicating with primary care (*SBAR*)

1. *Stop and Watch Early Warning Tool*: Care assistants or nurses will use this when they or anyone else in the nursing home (including other staff and care partners) notices a change in a resident, at latest by the end of the shift. They will circle observed changes, notifying the

For peer review only - http://bmjopen.bmj.com/site/about/guidelines.xhtml

For peer review only

nurse and giving them the completed tool to be placed in the nursing home records.

2. *Care Pathway*: this is a two-step clinical guidance and decision support system. The initial "Primary" assessment comprises screening questions with the potential to trigger a more detailed "Secondary" assessment. If the Primary or Secondary Assessment result is ambiguous, the *Care Pathway* will be administered at 6-hour intervals, until concerns have resolved and/or appropriate intervention has been instigated. The nurse will record the outcome of the Primary and Secondary Assessment and their care plan in the residents' care records and make a clinical decision about the next course of action which may include direct further monitoring using the *Stop and Watch Early Warning Tool*, initiating treatment in the nursing home, or if the assessment indicates a potential diagnosis or immediate concern, communication with primary care using the *SBAR* process. The nurse will feed back on the course of action to relevant staff on each shift, domestic staff and care partners, as appropriate. Copies of the completed *Care Pathway* will be kept with the resident's record.
3. *The SBAR (Situation, Background, Assessment, and Recommendation) method:* A structured method for communicating critical information to primary care. The nurse uses this when they want primary care input for one of their residents after the *Care Pathway* indicates a risk of decline. Before calling, the nurse will organise the briefing information on paper using the four elements (Situation, Background, Assessment and Recommendation).

Usual care group

Participants in nursing homes randomised to control arms will receive usual care according to existing local policy and practice. All medications and treatments will be permitted.

Study measures

We will collect data in three domains; 1) individual level data on nursing home residents, their care partners and staff, 2), system-level data, and 3) process data.

Individual-level data

These will be collected from staff, care partners or residents who have given informed consent or from residents for whom we have obtained agreement from a consultee. These data includes resident, care partner and staff demographics, assessment of resident functional status (Barthel index[33]) and resident service use and quality of life to inform health economic analysis (Client Services Receipt Inventory[34] (CSRI), EQ-5D-5L[35])). Staff education, role and the extent to which they perceive the organisation to support person centred care (Organisational Support for Person-centred Care[36] (P-CAT)) and Nurse Ratings of Communication with Primary Care Questionnaire[37] will be gathered to understand the context (table 2).

For peer review only - http://bmjopen.bmj.com/site/about/guidelines.xhtml

System-level data

These will be collected from the nursing home using existing paper or electronic record systems. Data will be pseudo-anonymised and provided by the nursing home manager or Research Facilitator. We will document the total number of contacts with GPs, ambulances, A&E visits, deaths, staff turnover and the number of nursing home beds available to new residents in the previous month, per care home. We will document all hospital admissions and select a sample of 30 of these (where we have individual consent from a resident to access their health records), purposively selected to represent a range of underlying ACSCs diagnoses. We will explore the "avoidability" of these with the Structured Implicit Record Review (SIRR) tool. [38] This facilitates comparison by two independent clinical experts (geriatrician and community nurse) of selected content in residents' nursing home records to assess whether the admission was "avoidable".

Process evaluation

We will examine how the intervention is implemented in practice, collecting information on the number of "Stop and Watch" Early Warning Tools completed, who noticed the change in the resident's health status, who completed the form, and actions that occurred after the form was completed; number of primary and secondary assessments, and outcome of assessments including the number of residents who underwent further monitoring, had their treatment initiated in the nursing home, or were referred to primary care. We will monitor intervention fidelity noting where nursing homes make amendments to the structure or content of the care pathway. PDCs will have a monthly support phone call with the research team to reflect on their activities and achievements which we will document. They will keep an activity log of support provided by Practice Development Support Groups to document the level of facilitation required to support the implementation process. To understand the effectiveness of study procedures we will collect data on consent and recruitment rates, the numbers of care partners who wish to be involved in the resident's care, assess completeness of outcome measures, data collection and return rate of questionnaires.

We will conduct 20 semi-structured interviews (30 to 45 minutes each) with 5 nursing home managers, 5 nurses, 5 care assistants and 5 care partners from the 7 intervention homes. We will explore participants' views on the effectiveness of the intervention in preventing avoidable hospital admissions and their experiences of implementing the intervention. Care partners will be purposively sampled to ensure a range of gender, age and types of family carer. All participants will give informed consent, including for recording of their interviews.

Data management

All data will be entered onto paper case report forms (CRFs) and then into an encrypted password protected database in accordance with the UK Data Protection Act and General Data Protection

For peer review only

Regulation (GDPR). CRFs will not bear the participant's name but a pseudo-anonymised identification number. To maintain confidentiality the Research Facilitator will remove the name of the participant from completed Stop and Watch Early Warning Tool and Care Pathway Primary and Secondary Assessment Forms and replace this with an ID number. Prior to analysis we will follow a standardised process for database lock.

Randomisation

The SAS statistical programme will be used for randomisation. Blinding is not feasible for research staff collecting data, but statisticians and health economists will be blinded to allocation. The randomisation variable will be supplied to them unlabelled, and main analysis completed using this.

Data analysis plan

We shall follow CONSORT guidelines for reporting randomised trials. Given this is a pilot study, analyses will be mainly descriptive focussing on recruitment, participant characteristics, other baseline and outcome variables, loss to follow-up and tabulation of serious adverse events. We shall compare rates of hospital admission for ACSCs and other outcomes between the control and intervention groups and calculate 95% confidence intervals. These, along with estimates of standard deviations of other outcome measures and intraclass correlation coefficients (ICCs) will inform the sample size calculation for a full trial. We will summarise completeness of data collection on outcome measures and, for questionnaires, describe distributions and response rates.

Economic evaluation

Analyses will conform to accepted economic evaluation methods (NICE, 2008). We will calculate costs associated with the intervention, including the cost of enhancing staff's knowledge and skills and resources associated with implementation. Resource use associated with hospital admissions, primary care and other NHS and social care costs will be collected using the CSRI. Costs will be reported from an NHS/PSS, government and societal perspective. We will assess feasibility of calculating quality adjusted life years (QALYs) for residents, using the EQ-5D- 5L. In the societal level analysis, a calculation of carers' QALYs will be included. We will provide an initial estimate of the incremental mean cost per QALY gained in intervention compared to control homes. Cost-effectiveness acceptability curves, showing the percentage of cases that the intervention is cost-effective over a range of values of willingness to pay for a QALY gained, will be constructed for each different costing perspective and for the different methods of calculating QALYs. We will model the lifetime costs and outcomes of the intervention compared to controls. This will involve assessing the quality of the published information available, the development of an initial model and identification of which cost and outcome components would benefit most from further research i.e. extra value of perfect information (EVPI) and extra value of partial perfect information (EVPPI) analysis.[39 40]

For peer review only - http://bmjopen.bmj.com/site/about/guidelines.xhtml

Qualitative methods

A verbatim transcript of qualitative interviews will be made and entered into qualitative analysis software (NVIVO) and key themes coded using framework analysis.[41] A sample of interviews will be analysed by two senior investigators to check levels of coding agreement with the template.

Public and patient involvement

The original research proposal was developed in collaboration with UK Dementia and Neurodegenerative Diseases Network (DeNDRoN) Patient and Public Involvement representatives (Nurock and Woodward-Carlton), who are grant co-applicants. Two Carer Reference Panels (CRPs), have been created to ensure public involvement at all stages, chaired by Nurock (London) and Woodward-Carlton (Bradford). Each panel comprises up to eight family carers of people with dementia and a person living with dementia. The carers are members of, and supported by, the Alzheimer's Society research volunteer network. They will meet at 6-monthly intervals throughout the programme, advise and work collaboratively on recruitment and consent processes, accessibility of information leaflets, data collection, interpretation and dissemination. Our CRP chairs will attend study recruitment and information events at participating care homes to publicise the trial. They provide strategic oversight by attending programme steering group and international advisory meetings. We held informal consultations with a group of seven residents living in one nursing home to inform development of study information materials for residents.

Monitoring and trial management

Our intervention is low-risk and an enhancement of usual care. We will collect data on potential serious adverse events (SAEs); those that may result in death, be life-threatening, require hospitalisation or prolongation of existing hospitalisation or result in persistent or significant disability or incapacity. These will be reported to the Chief Investigator and escalated to the research ethics committee if they are unexpected and related to research procedures.

A Trial Management Group meets monthly and includes individuals responsible for day-to-day study management. It will monitor all aspects of the conduct and progress of the study, ensuring adherence to the protocol and will take appropriate action to safeguard participants and trial quality. For this pilot trial it also takes on the role of a Data Monitoring Committee. The BHiRCH Programme Steering Committee includes senior members of the research team and external experts and supervises the overall programme, on behalf of NIHR and the Sponsor. An International Advisory Group (IAG) provides advice and guidance to ensure the project remains grounded in real experience and is informed by international best practice and research.

For peer review only - http://bmjopen.bmj.com/site/about/guidelines.xhtml

DISCUSSION

The aim of our study is to pilot test an intervention to reduce avoidable acute hospital admissions for the four most common ACSCs in UK nursing home residents. Our intervention is closely aligned with UK health policy which aims to improve healthcare provided within nursing homes and prevent distressing transitions to acute hospitals. Despite policy imperatives, little empirical research has been conducted in this area in the UK. The quality of intervention studies has varied with insufficient attention paid to methodology, particularly implementation, intervention adherence and clustering effects.

We have adapted elements of an existing intervention (INTERACT) to the UK context with stakeholder input and extensive co-design. Trials of complex interventions are often methodologically challenging but the nursing home environment brings additional issues. Each nursing home, even those that are part of large company chains, is unique in context, with a distinct culture of care and variability in practice which may be greater than that seen in clinical NHS settings. A particular strength of our intervention is that the implementation is informed by implementation science theory, tailored to the context and led by local Practice Development Champions supported by a local Practice Development Champion Support Group comprised of key stakeholders who can assist with achieving change in care practice.

We anticipate participant recruitment, particularly of residents with impaired capacity to consent to research procedures may be challenging. Additionally, nursing homes may not have the infra-structure to support data collection data. We plan to overcome these problems through developing the role of an existing member of the nursing home staff as a Research Facilitator and will provide reimbursement for their time and additional work.

Our pilot study will provide data to indicate whether a fully-powered randomised controlled trial is warranted. In addition it will provide invaluable information on how to optimise the implementation of complex interventions in nursing homes.

ETHICS AND DISSEMINATION

The trial is approved by London Queen Square Research Ethics Committee (Reference: 17/LO/1542) and the UK Health Research Authority (HRA). Some residents may have dementia or other conditions that impair their ability to give informed consent to participate in the individual data collection and we will adhere to the UK Mental Capacity Act (2005). We have an established procedure should we observe incidents of concern and will adhere to local authority safeguarding procedures.

For peer review only - http://bmjopen.bmj.com/site/about/guidelines.xhtml

Academic dissemination will be through publication of results, reported per CONSORT guidelines in international peer-review journals and conference presentations. Regular updates will be given via social media, our website (https://www.brad.ac.uk/health/dementia/research/bhirch/) and twitter (@BHIRCHCareHomes). We will present at congresses attended by nursing home owners and staff and will hold a national conference at the end of the study to disseminate findings and share best practice on active care for ACSCs.

For peer review only

Acknowledgements

The authors would like to thank the members of our carer reference panels and the Alzheimer's Society research network volunteers. We are grateful to the nursing homes that supported our feasibility study.

Authors' contributions

ELS, AF, AB, KF, RH, LM, BMcC, SN, MP, CP, GR, LR, BW-C, JY, MD were involved in development of the intervention and made substantial contributions to the concept and design of the study and protocol. ELS and AF wrote the protocol paper. ELS, AF, AB, KF, RH, LM, BMcC, SN, MP, CP, GR, LR, BW-C, JY, MD critically revised the manuscript and approved the final version.

Funding: This work was supported by the UK NIHR grant number RP-PG-0612-20010. The views expressed are those of the author(s) and not necessarily those of the NHS, the NIHR or the Department of Health and Social Care.

Competing interests statement: none declared

Ethics approval: approved by London Queen Square Research Ethics Committee (Reference: 17/LO/1542)

Provenance and peer review: not commissioned; externally peer reviewed

For peer review only

References

1. Laing and Buisson. Care of Older People UK Market Report. 28th ed, 2017.

2. British Geriatric Society. Quest for quality in care homes: British Geriatrics Society Joint Working Party Inquiry into the Quality of Healthcare Support for Older People in Care Homes: A Call for Leadership, Partnership and Quality Improvement. London: British Geriatric Society, 2011.

3. Dudman J, Meyer J. Understanding residential home issues to meet health-care needs. *British Journal of Community Nursing* 2012;17(9):434-8.

4. McDougall F, Matthews F, Kvaal K, et al. Prevalence and symptomatology of depression in older people living in institutions in England and Wales. *Age and Ageing* 2007;36(5):562-8.

5. Baylis A, Perks-Baker S. Enhanced health in care homes: Learning from experiences so far. London, 2017.

6. Carter MW, Porell FW. Vulnerable populations at risk of potentially avoidable hospitalizations: the case of nursing home residents with Alzheimer's disease. *American journal of Alzheimer's disease and other dementias* 2005;20(6):349-58. [published Online First: 2006/01/07]

7. Grabowski DC, O'Malley AJ, Barhydt NR. The costs and potential savings associated with nursing home hospitalizations. *Health affairs (Project Hope)* 2007;26(6):1753-61. doi: 10.1377/hlthaff.26.6.1753 [published Online First: 2007/11/06]

8. Tian Y, Dixon A, Gao H. Emergency hospital admissions for ambulatory care-sensitive conditions: identifying the potential for reductions: King's Fund, 2012.

9. Bardsley M, Blunt I, Davies S. Is secondary preventive care improving? Observational study of 10-year trends in emergency admissions for conditions amenable to ambulatory care. *BMJ Open* 2013;3(1) doi: 10.1136/bmjopen-2012- 002007

For peer review only - http://bmjopen.bmj.com/site/about/guidelines.xhtml

10. HES Analysis Team. Counts of finished admission episodes (FAEs) with an emergency admission by source of admission from 2009-10 to 2015-16. In: Health and Social Care Information Centre, ed. Leeds, 2016.

11. Purdy S, Griffin T, Salisbury C, et al. Ambulatory care sensitive conditions: terminology and disease coding need to be more specific to aid policy makers and clinicians. *Public Health* 2009;123:169-73.

12. Walsh E, Wiener J, Haber S. Potentially avoidable hospitalizations of dually eligible Medicare and Medicaid beneficiaries from nursing facility and Home- and Community-Based Services waiver programs. *Journal of the American Geriatric Society* 2012;60(5):821-9. doi: 10.1111/j.1532-5415.2012.03920.x

13. Carter M, Porell F. Vulnerable populations at risk of potentially avoidable hospitalizations: the case of nursing home residents with Alzheimer's disease. *American Journal of Alzheimer's Disease and Other Dementias* 2005;20(6):349-58.

14. Krüger K, Jansen K, Grimsmo A. Hospital admissions from nursing homes: Rates and reasons. *Nursing Research and Practice* 2011 doi: 10.1155/2011/247623

15. Ouslander J, Diaz S, Hain D. Frequency and diagnoses associated with 7- and 30- day readmission of skilled nursing facility patients to a nonteaching community hospital. *Journal of the American Medical Directors Association* 2011;12(3):195-203. doi: 10.1016/j.jamda.2010.02.015

16. Ouslander J, Lamb G, Tappen R. Interventions to reduce hospitalizations from nursing homes: evaluation of the INTERACT II collaborative quality improvement project. *Journal of the American Geriatric Society* 2011;59(4):745-53. doi: 10.1111/j.1532-5415.2011.03333.x

17. Alessi C, Harker J. A prospective study of acute illness in the nursing home. *Aging* 1998;10(6):479-89.

18. Mukadam N, Sampson EL. A systematic review of the prevalence, associations and outcomes of dementia in older general hospital inpatients. *IntPsychogeriatr* 2010:1-12.

For peer review only - http://bmjopen.bmj.com/site/about/guidelines.xhtml

19. Watkin L, Blanchard MR, Tookman A, et al. Prospective cohort study of adverse events in older people admitted to the acute general hospital: risk factors and the impact of dementia. *IntJGeriatrPsychiatry* 2012;27(1):76-82. doi: 10.1002/gps.2693 [doi]

20. Smith P, Sherlaw-Johnson C, Ariti C, et al. Quality watch. Focus on: Hospital admissions from care homes. London: Health Foundation and Nuffield Trust, 2015.

21. National Health Service. NHS Outcomes Framework 2016 to 2017, 2016.

22. Ouslander JG, Perloe M, Givens JH, et al. Reducing potentially avoidable hospitalizations of nursing home residents: results of a pilot quality improvement project. *JAmMedDirAssoc* 2009;10(9):644-52. doi: S1525-8610(09)00248-5 [pii];10.1016/j.jamda.2009.07.001 [doi]

23. Ouslander JG, Berenson RA. Reducing unnecessary hospitalizations of nursing home residents. *NEnglJMed* 2011;365(13):1165-67. doi: 10.1056/NEJMp1105449 [doi]

24. Lisk R, Yeong K, Nasim A, et al. Geriatrician input into nursing homes reduces emergency hospital admissions. *Archives of gerontology and geriatrics* 2012;2011/11/25:331-37.

25. Codde J, Arendts G, Frankel J, et al. Transfers from residential aged care facilities to the emergency department are reduced through improved primary care services: an intervention study. *Australasian journal on ageing* 2010;2010/12/15:150-54.

26. Kane RL, Keckhafer G, Flood S, et al. The effect of Evercare on hospital use. *Journal of the American Geriatrics Society* 2003;2003/09/27:1427-34.

27. Mor V, Intrator O, Fries B. Changes in hospitalization associated with introducing the Resident Assessment Instrument. *Journal of the American Geriatric Society* 1997;45(8):1002-10.

28. Young Y, Inamdar S, Dichter B. Clinical and nonclinical factors associated with potentially preventable hospitalizations among nursing home residents in New York State. *Journal of the American Medical Directors Association* 2011;12(5):364-71. doi: 10.1016/j.jamda.2010.03.006

29. Ouslander J, Perloe M, Givens J. Reducing potentially avoidable hospitalizations of nursing home residents: results of a pilot quality improvement project. *Journal of the*

For peer review only - http://bmjopen.bmj.com/site/about/guidelines.xhtml

American Medical Directors Association 2009;10(9):644-52. doi: 10.1016/j.jamda.2009.07.001

30. Olsan T. Reducing avoidable Rehospitalisations by Improving Residents' Transitions to the Nursing Home. 2011:B22.

31. Kitson AL, Rycroft-Malone J, Harvey G, et al. Evaluating the successful implementation of evidence into practice using the PARiHS framework: theoretical and practical challenges. *ImplementSci* 2008;3:1.

32. Rycroft-Malone J, Harvey G, Seers K, et al. An exploration of the factors that influence the implementation of evidence into practice. *JClinNurs* 2004;13(8):913-24.

33. Mahoney FI, Barthel DW. Functional Evaluation: The Barthel Index. *Md State Med J* 1965;14:61-5. [published Online First: 1965/02/01]

34. Beecham J, Knapp M. Costing psychiatric interventions. In: Thornicroft G, ed. Measuring Mental Helath Needs. London: Gaskell 2001:200-24.

35. Herdman M, Gudex C, Lloyd A, et al. Development and preliminary testing of the new five-level version of EQ-5D (EQ-5D-5L). *Qual Life Res* 2011;20(10):1727-36. doi: 10.1007/s11136-011-9903-x [published Online First: 2011/04/12]

36. Edvardsson D, Fetherstonhaugh D, Nay R, et al. Development and initial testing of the Person-centered Care Assessment Tool (P-CAT). *International psychogeriatrics / IPA* 2010;22(1):101-8. doi: 10.1017/S1041610209990688 [published Online First: 2009/07/28]

37. Tjia J, Mazor KM, Field T, et al. Nurse-physician communication in the long-term care setting: perceived barriers and impact on patient safety. *J Patient Saf* 2009;5(3):145-52. doi: 10.1097/PTS.0b013e3181b53f9b [published Online First: 2009/11/21]

38. Saliba D, Kington R, Buchanan J, et al. Appropriateness of the decision to transfer nursing facility residents to the hospital. *Journal of the American Geriatrics Society* 2000;2000/02/22:154-63.

39. Ades AE, Lu G, Claxton K. Expected value of sample information calculations in medical decision modeling. *Med Decis Making* 2004;24(2):207-27. doi: 10.1177/0272989X04263162 [published Online First: 2004/04/20]

For peer review only - http://bmjopen.bmj.com/site/about/guidelines.xhtml

40. Coyle D, Oakley J. Estimating the expected value of partial perfect information: a review of methods. *Eur J Health Econ* 2008;9(3):251-9. doi: 10.1007/s10198-007-0069-y [published Online First: 2007/07/20]

41. Ritchie J, Spencer L. Qualitative data analysis for applied policy research. In: Ryman A, Burgess AG, eds. Analysing qualitative data. London: Routledge 1994.

For peer review only

Table 1. Practice Development Champion person specification

The Practice Development Champion will:<br>• Be a Registered Nurse.<br>• Have been working in the nursing home for at least 6 months.<br>When selecting a Practice Development Champion we are looking for someone who:<br>• Has some knowledge of good practice in supporting health care and has an interest in the topic *(can demonstrate some essential knowledge of the management of the 4 conditions: chronic heart failure, respiratory infections, UTI, dehydration).*<br>• Knows co-workers *(has been in the organisation long enough to know the staff and how they work).*<br>• Knows the environment *(has some insight into the culture of the setting).*<br>• Knows the organisation *(knows their way around the organisation, e.g. who's who, policies in place, decision-making structures).*<br>• Possesses effective communication skills *(could include attributes of being open minded, being creative, has experience of managing meetings/groups, able to talk in front of groups).*<br>• Is self-aware and resilient *(has insight into their support needs, but is also not afraid of challenge/conflict; willing to engage in own professional development).*<br>• Is reliable and dependable *(has time they can dedicate to this work [in writing from their manager]; carries through with responsibilities, meets deadlines or negotiates otherwise; is not intending to be on extended leave during intervention period).*

- Is respected by co-workers (*has a good relationship with co-workers which means they will be listened to with respect to new ideas*).

These criteria are ESSENTIAL and are NOT listed in a hierarchy/order of importance, i.e. they are all equally important.
From Seers et al 2012 FIRE (facilitating implementation of research evidence): a study protocol http://implementationscience.biomedcentral.com/articles/10.1186/1748-5908-7-25

For peer review only

BHiRCH pilot RCT protocol paper

Table 2. Summary of data collected, outcome measures and time schedule

	Data collected and tool used	***Pre-intervention***	***Monthly***	***At 6 months only***	***Post intervention***
Resident					
Socio-demographics	Age, gender, ethnicity, marital status, highest level of education	R	-	-	-
Service use in the prior month	Client Service Receipt Inventory (CSRI) (ref). Calculates service and total care costs	R	R	R	R
Functional status	The Barthel Index [33]	R	-	R	R
Resident quality of life-self rated	EQ-5D-5L (ref) self-rated health index and visual analogue scale of current health state.	P	-	P	P
Resident quality of life-proxy rated	EQ-5d-Proxy (ref) care partner or staff member view of the resident's QoL	CP/S	-	CP/S	CP/S
Care partner					
Socio-demographics	Age, gender, ethnicity, marital status, years of schooling, highest level of education.	CP	-	CP	CP
Quality of life	EQ-5D-5L EuroQol (1990)	CP	-	CP	CP
Preferred role	How much and how they like to be involved in the residents care	CP	-	-	-
Staff					
Staff socio-demographics	Age, gender, ethnicity, number of years of education,	R	-	-	-
Staff work characteristics	Highest qualification, role in care home, length of service, shift pattern, first language	R	-	-	-
Organizational support for person-centered care	The Person-Centred Care Assessment Tool (P-CAT) (ref)	S	-	S	S
Communication with primary care	Nurse-GP Communication Needs Assessment Questionnaire	S	-	S	S
Perceived knowledge and skills for early detection in changes in health	Developed from feasibility study. Assesses key knowledge and skills needed to implement the intervention. Rated on 5-point likert scale; 1 (disagree completely) to 5 (agree completely).	S	-	S	S
**System-level data**					
Number of hospital admissions	Respiratory infections, urinary tract infections, dehydration, congestive heart failure?	S	S	S	S
"Avoidability" of admissions	Structured Implicit Record Review (SIRR; Saliba et al., 2000)	S	S	S	S
Use of Primary assessment tool	Respiratory infections, urinary tract infections, dehydration, congestive heart failure?	S	S	S	S
Use of Secondary assessment	Respiratory infections, urinary tract infections, dehydration, congestive heart failure?	S	S	S	S
Out of hours GP contacts	GP visits or telephone contact	S	S	S	S
Ambulances and hospital use	Number and length of hospital admissions (days) , A&E attendances and readmissions	S	S	S	S
Deaths in the last calendar month		S	S	S	S
Staff turnover		S	S	S	S
Care home occupancy level	Number of available beds to new residents	S	S	S	S

Measure assessed by: P, participant; CP, care partner; R researcher; S, care home staff.

For peer review only - http://bmjopen.bmj.com/site/about/guidelines.xhtml

Legend for figure

Figure 1. Pilot Trial Flow Chart

For peer review only

For peer review only - http://bmjopen.bmj.com/site/about/guidelines.xhtml

Figure 1: Pilot Trial Flow Chart

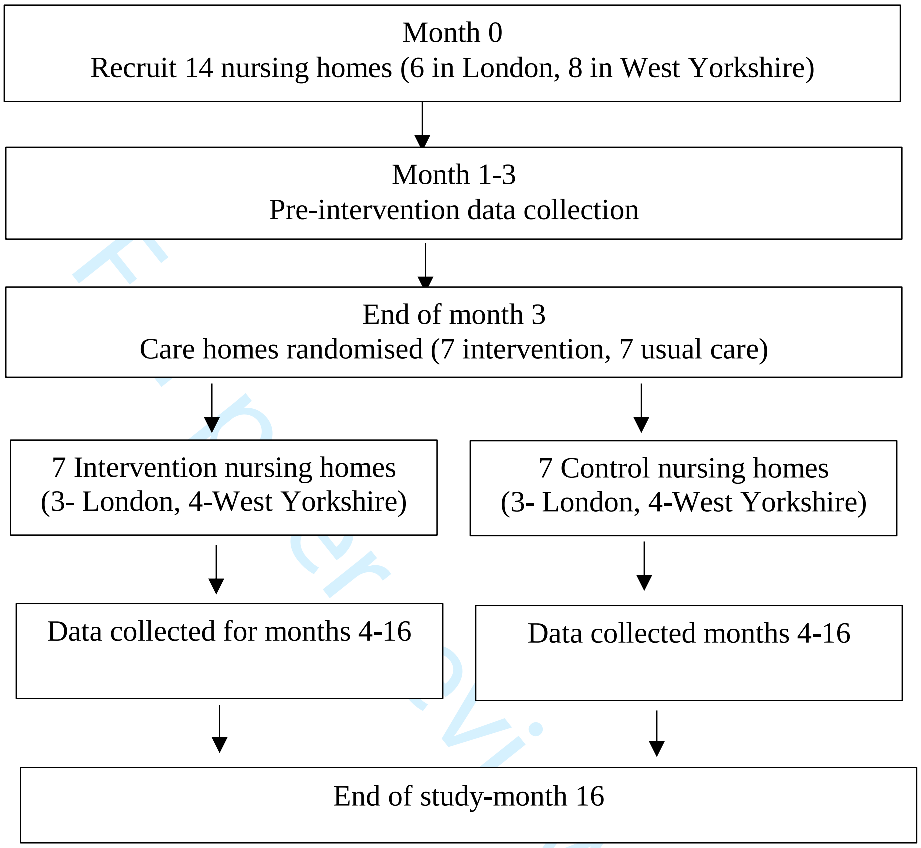

For peer review only

STANDARD PROTOCOL ITEMS: RECOMMENDATIONS FOR INTERVENTIONAL TRIALS

BHiRCH
intervention protocol-paper

SPIRIT 2013 Checklist: Recommended items to address in a clinical trial protocol and related documents*

Section/item	Item No	Description
**Administrative information**		
Title	1	Descriptive title identifying the study design, population, interventions, and, if applicable, trial acronym -yes
Trial registration	2a	Trial identifier and registry name. If not yet registered, name of intended registry-yes
	2b	All items from the World Health Organization Trial Registration Data Set-on protocol
Protocol version	3	Date and version identifier-yes uploaded
Funding	4	Sources and types of financial, material, and other support-yes
Roles and responsibilities	5a	Names, affiliations, and roles of protocol contributors-yes
	5b	Name and contact information for the trial sponsor-on protocol
	5c	Role of study sponsor and funders, if any, in study design; collection, management, analysis, and interpretation of data; writing of the report; and the decision to submit the report for publication, including whether they will have ultimate authority over any of these activities-on protocol
	5d	Composition, roles, and responsibilities of the coordinating centre, steering committee, endpoint adjudication committee, data management team, and other individuals or groups overseeing the trial, if applicable (see Item 21a for data monitoring committee)-yes
**Introduction**		
Background and rationale	6a	Description of research question and justification for undertaking the trial, including summary of relevant studies (published and unpublished) examining benefits and harms for each intervention-yes
	6b	Explanation for choice of comparators-yes
Objectives	7	Specific objectives or hypotheses-yes

For peer review only

Trial design	8	Description of trial design including type of trial (eg, parallel group, crossover, factorial, single group), allocation ratio, and framework (eg, superiority, equivalence, noninferiority, exploratory)-yes

Methods: Participants, interventions, and outcomes

Study setting	9	Description of study settings (eg, community clinic, academic hospital) and list of countries where data will be collected. Reference to where list of study sites can be obtained-yes
Eligibility criteria	10	Inclusion and exclusion criteria for participants. If applicable, eligibility criteria for study centres and individuals who will perform the interventions (eg, surgeons, psychotherapists)-yes
Interventions	11a	Interventions for each group with sufficient detail to allow replication, including how and when they will be administered-yes referenced
	11b	Criteria for discontinuing or modifying allocated interventions for a given trial participant (eg, drug dose change in response to harms, participant request, or improving/worsening disease)- not relevant
	11c	Strategies to improve adherence to intervention protocols, and any procedures for monitoring adherence (eg, drug tablet return, laboratory tests)-yes
	11d	Relevant concomitant care and interventions that are permitted or prohibited during the trial-yes
Outcomes	12	Primary, secondary, and other outcomes, including the specific measurement variable (eg, systolic blood pressure), analysis metric (eg, change from baseline, final value, time to event), method of aggregation (eg, median, proportion), and time point for each outcome. Explanation of the clinical relevance of chosen efficacy and harm outcomes is strongly recommended-yes
Participant timeline	13	Time schedule of enrolment, interventions (including any run-ins and washouts), assessments, and visits for participants. A schematic diagram is highly recommended (see Figure)-yes
Sample size	14	Estimated number of participants needed to achieve study objectives and how it was determined, including clinical and statistical assumptions supporting any sample size calculations-yes
Recruitment	15	Strategies for achieving adequate participant enrolment to reach target sample size-yes

Methods: Assignment of interventions (for controlled trials)

Allocation:

Sequence generation	16a	Method of generating the allocation sequence (eg, computer-generated random numbers), and list of any factors for stratification. To reduce predictability of a random sequence, details of any planned restriction (eg, blocking) should be provided in a separate document that is unavailable to those who enrol participants or assign interventions-yes
Allocation concealment mechanism	16b	Mechanism of implementing the allocation sequence (eg, central telephone; sequentially numbered, opaque, sealed envelopes), describing any steps to conceal the sequence until interventions are assigned-not relevant
Implementation	16c	Who will generate the allocation sequence, who will enrol participants, and who will assign participants to interventions-yes
Blinding (masking)	17a	Who will be blinded after assignment to interventions (eg, trial participants, care providers, outcome assessors, data analysts), and how-yes
	17b	If blinded, circumstances under which unblinding is permissible, and procedure for revealing a participant's allocated intervention during the trial-yes
**Methods: Data collection, management, and analysis**		
Data collection methods	18a	Plans for assessment and collection of outcome, baseline, and other trial data, including any related processes to promote data quality (eg, duplicate measurements, training of assessors) and a description of study instruments (eg, questionnaires, laboratory tests) along with their reliability and validity, if known. Reference to where data collection forms can be found, if not in the protocol-yes
	18b	Plans to promote participant retention and complete follow-up, including list of any outcome data to be collected for participants who discontinue or deviate from intervention protocols-yes
Data management	19	Plans for data entry, coding, security, and storage, including any related processes to promote data quality (eg, double data entry; range checks for data values). Reference to where details of data management procedures can be found, if not in the protocol-yes
Statistical methods	20a	Statistical methods for analysing primary and secondary outcomes. Reference to where other details of the statistical analysis plan can be found, if not in the protocol-yes
	20b	Methods for any additional analyses (eg, subgroup and adjusted analyses)-yes
	20c	Definition of analysis population relating to protocol non-adherence (eg, as randomised analysis), and any statistical methods to handle missing data (eg, multiple imputation)-yes

Methods: Monitoring

Data monitoring	21a	Composition of data monitoring committee (DMC); summary of its role and reporting structure; statement of whether it is independent from the sponsor and competing interests; and reference to where further details about its charter can be found, if not in the protocol. Alternatively, an explanation of why a DMC is not needed-yes
	21b	Description of any interim analyses and stopping guidelines, including who will have access to these interim results and make the final decision to terminate the trial-not relevant
Harms	22	Plans for collecting, assessing, reporting, and managing solicited and spontaneously reported adverse events and other unintended effects of trial interventions or trial conduct-yes
Auditing	23	Frequency and procedures for auditing trial conduct, if any, and whether the process will be independent from investigators and the sponsor-in protocol

Ethics and dissemination

Research ethics approval	24	Plans for seeking research ethics committee/institutional review board (REC/IRB) approval-yes
Protocol amendments	25	Plans for communicating important protocol modifications (eg, changes to eligibility criteria, outcomes, analyses) to relevant parties (eg, investigators, REC/IRBs, trial participants, trial registries, journals, regulators)-yes
Consent or assent	26a	Who will obtain informed consent or assent from potential trial participants or authorised surrogates, and how (see Item 32)-yes
	26b	Additional consent provisions for collection and use of participant data and biological specimens in ancillary studies, if applicable-not relevant
Confidentiality	27	How personal information about potential and enrolled participants will be collected, shared, and maintained in order to protect confidentiality before, during, and after the trial-yes
Declaration of interests	28	Financial and other competing interests for principal investigators for the overall trial and each study site-yes
Access to data	29	Statement of who will have access to the final trial dataset, and disclosure of contractual agreements that limit such access for investigators-yes
Ancillary and post-trial care	30	Provisions, if any, for ancillary and post-trial care, and for compensation to those who suffer harm from trial participation-in protocol

For peer review only

Dissemination policy	31a	Plans for investigators and sponsor to communicate trial results to participants, healthcare professionals, the public, and other relevant groups (eg, via publication, reporting in results databases, or other data sharing arrangements), including any publication restrictions-yes
	31b	Authorship eligibility guidelines and any intended use of professional writers-not relevant
	31c	Plans, if any, for granting public access to the full protocol, participant-level dataset, and statistical code-not relevant
**Appendices**		
Informed consent materials	32	Model consent form and other related documentation given to participants and authorised surrogates-in protocol
Biological specimens	33	Plans for collection, laboratory evaluation, and storage of biological specimens for genetic or molecular analysis in the current trial and for future use in ancillary studies, if applicable-not relevant

*It is strongly recommended that this checklist be read in conjunction with the SPIRIT 2013 Explanation & Elaboration for important clarification on the items. Amendments to the protocol should be tracked and dated. The SPIRIT checklist is copyrighted by the SPIRIT Group under the Creative Commons "Attribution-NonCommercial-NoDerivs 3.0 Unported" license.

For peer review only
